# Discovery of natural scaffolds as HER2 inhibitors for breast cancer: virtual screening, molecular dynamics, and biological characterization with selectivity profiling

**DOI:** 10.1038/s41598-025-11177-6

**Published:** 2025-07-17

**Authors:** Asmaa Hossam, Ingy I. Abdallah, Nadia A. El-Sebakhy, Radwan Alnajjar, Mohamed M. Mohyeldin

**Affiliations:** 1 Department of Pharmacognosy, Faculty of Pharmacy, Alexandria University, Alexandria, 21521 Egypt; 2 Department of Chemistry, Faculty of Science, University of Benghazi, Benghazi, Libya

**Keywords:** Breast cancer, Virtual screening, HER2, Western blot, Molecular dynamics, Liquiritin, Cancer, Drug discovery

## Abstract

Nature offers potential therapeutic candidates for breast cancer (BC), and targeting HER2 signaling presents a promising approach, leveraging the successful history of anticancer drug discovery. Using a structure-based virtual screening workflow, a comprehensive library of natural products (NPs) was screened for potential HER2 binding. Five of these NPs were selected for in-depth biological validation against BC. Biochemically, oroxin B, liquiritin, ligustroflavone, and mulberroside A suppressed HER2 catalysis with nanomolar potency. Binding mode studies of NPs revealed their binding patterns, providing valuable SAR insights for effective HER2 inhibition. Further cellular assays revealed that the top NPs have preferential anti-proliferative effects towards HER2 over-expressing BC cells, with notable selectivity indices. Liquiritin exhibited promising anti-migratory activity in two cellular motility models, while other tested hits primarily inhibited cancer cell growth with minimal effects on metastasis. Liquiritin and oroxin B stood out as validated hits, revealing the most promising profiles. ADME predictions and MD simulations positioned liquiritin as a more promising HER2 inhibitor than oroxin B, despite oroxin B’s higher ranking in rigid docking studies. On the molecular level, liquiritin significantly inhibited HER2 phosphorylation and expression in BC cells. Liquiritin demonstrated notable selectivity for HER family proteins when tested against various kinases, highlighting its potential as a pan-HER inhibitor hit for future development. Further in vivo assessment is necessary to support the hit-to-lead promotion of liquiritin.

**Supplementary Information:**

The online version contains supplementary material available at 10.1038/s41598-025-11177-6.

## Introduction

Breast cancer is the most frequently diagnosed cancer in women worldwide. HER2-positive breast cancer is the second most aggressive subtype of breast cancer, with a poor prognosis and survival rate, making up to one-third of all breast cancer diagnoses^1^. HER2 is considered an inherent member of the epidermal growth factor receptor family (HER/EGFR/ERBB) and portrays crucial functions in cell survival, proliferation, and resistance to apoptosis^2^. HER2-positive malignancies are defined as having abnormal HER2 protein expression or unregulated tyrosine kinase activation, which enhances carcinogenic processes such as tumor genesis, progression, and aggressiveness^3^. The protein known as the HER2 receptor has an extracellular domain and a Tyrosine Kinase (TK) domain. A promising treatment approach is the functional inhibition of the enzyme through its extracellular domain or HER2-TK^4^. Currently, only very specialized therapeutic formulations based on the monoclonal antibody approach (Trastuzumab) binding to the extracellular domain or small molecule kinase inhibitors (Lapatinib) are found successful against tumors with HER2 phenotype, which are frequently resistant to standard chemotherapy. These treatment modalities provided promising results in treating HER2-positive malignancies^5^. Still, it has drawbacks such as toxicity and, in some situations, a gradual loss of sensitivity and failure of the targeted therapy^6^. Small molecule TK inhibitors are less costly and exhibit fewer side effects compared to monoclonal antibodies^7^. Therefore, this situation necessitates the development of novel and effective HER2-TK inhibitors with low toxicity profiles for use as targeted anti-cancer medications.

Natural products provide an efficient substitute and expanded repertoire for hit/lead discovery since they are reasonably priced, have fewer adverse effects, and are supported by a history of success in anticancer drug discovery^8^. To address the urgent need for new HER2 inhibitors, this study leverages an integrative in silico and in vitro pipeline to identify promising natural product candidates. We implemented a hierarchical virtual screening strategy using Glide HTVS/SP/XP docking for efficient initial screening of a large natural product library. Biochemically validated hits underwent retrospective induced fit docking to assess pose stability and receptor flexibility followed by further characterization through molecular dynamics simulations and MM-GBSA rescoring. This approach balanced computational efficiency against pose accuracy, leveraging progressive docking stringency to mitigate false positives while MM-GBSA addressed scoring uncertainties. Retrospective validation confirmed pose stability under induced fit conditions, supporting methodological robustness. Top-ranking compounds were further subjected to comprehensive in vitro validation, including a series of cellular assays, and Western blot analysis. This multi-stage workflow not only maximized computational efficiency and accuracy but also provided a rigorous, experimentally validated foundation for identifying novel HER2-targeting agents. Discovery of novel lead small molecules from natural sources targeting HER2 will open up new avenues for the targeted treatment of HER2-positive tumors.

## Materials and methods

### Molecular docking studies

#### In-house virtual library compilation, training set construction, and ligand preparation

The in-house library of almost three-quarters of a million natural products belonging to diverse chemical classes with a particular emphasis on chemical scaffolds with previously reported anticancer activity was compiled from nine commercial natural product databases as COCONUT (406,748 compounds), ZINC Natural Products Catalogue (270,549 compounds), SANCDB (1012 compounds), NPATLAS (29,006 compounds), NCI Natural Products Repository (1035 compounds), AFRONDP (1243 compounds), ANALYTICON MEGX (10,595 compounds), ANPDB (13,306 compounds) and ICC (3,180 compounds) databases. After the removal of duplicates, the 2D structures of 638.960 natural products were retrieved from the PubChem database (compiled natural products library is available in the supplementary files). Meanwhile, a training set was tailored to include standard specific HER2 inhibitors, dual HER2/other kinase inhibitors, pan-HER family inhibitors, as well as multikinase inhibitors with HER2 kinase as one of their molecular targets. The training set consisted of 18 standard HER2 kinase inhibitors satisfying one of the aforementioned criteria, including the two marketed HER2 inhibitors: lapatinib and neratinib, as shown in Table S1. The training set was implemented to validate the adopted virtual screening protocol. Ligands were prepared for virtual screening using the LigPrep 2.3 module of Schrödinger molecular modeling suite (LigPrep, version 2.3, 2018, Schrödinger).

#### HER2 protein preparation and grid generation

The X-ray crystal structure of TK domain of human HER2 protein in complex with N-[2-[4-([3 chloro-4-[3-(trifluoromethyl)phenoxy]phenyl]amino)-5H-pyrrolo[3,2-d]pyrimidin-5yl]ethyl]3-hydroxy-3-methylbutanamide (TAK-285) as an inhibitor (PDB ID: 3RCD) was chosen for structure-based virtual screening, and obtained in PDB file format from RCSB Protein Data Bank (http://www.rcsb.org). The preparation of the protein was executed via the Schrödinger suite’s protein preparation wizard module. Initially, the protein was preprocessed by removing water molecules 5 Å away from the active site, eliminating unwanted chains, filling out missing loops or side chains, adding hydrogens, capping uncapped C and N termini at pH 7 ± 2, amending bonds and formal charges for metals, creating disulfide bonds, and adjusting mislabeled elements. Following that, the network of hydrogen bonds and Asn, Gln, and His residues’ orientation in the protein were optimized by applying PROPKA at PH = 7 in order to improve charge-charge interactions and hydrogen bonding with neighboring groups. Lastly, a restricted minimization of the protein structure was accomplished utilizing OPLS 3 force field with root mean square displacement (RMSD) value of 0.3°A to relieve any strain and fine-tune the placement of various groups.

For docking experiments, the binding site was assigned using the receptor grid generating module. The receptor grid cubic box was established with dimensions of 20 × 20 × 20 Å around the co-crystallized ligand (TAK-285) centroid with a grid spacing of 0.375 Å. The receptor grid’s size was amended to fit ligands only of maximum size 20 Å for eliminating big molecules with overrated binding affinities.

#### **Molecular docking**

GLIDE enrichment calculator was applied to validate the sensitivity and specificity of the docking protocol for the prediction of geometric poses and calculation of the scores of protein-ligand interactions using the compiled training set. The enrichment calculator determines enrichment metrics for a screen that was run with a set of actives (a compiled training set) and a set of decoys. These metrics include, but are not limited to, ROC, AUC-ROC, BEDROC, and EF (at 2%, 5%, and 10%).

Following validation, using the Maestro Glide module (Glide, version 11.8, 2018, Schrodinger, USA), the virtual screening campaign was carried out between the optimized ligands and the ATP binding site of the HER2 TK domain in three successive stages of molecular docking. Initially, high-throughput virtual screening (HTVS) of the compiled natural products database was carried out through the HTVS mode in the Glide module of Maestro. Following the HTVS simulation, docking scores and binding poses produced were used to rank the resulting hits and keep only those with reasonable docking scores (≥ -6.00 kcal/mol). Based on HTVS results, the top 10,000 natural products with promising HER2 binding affinities were selected for further investigation. At first, docking of those compounds into the active site of HER2 protein was performed using standard precision (SP) glide mode. Next, the top 500 among these 10,000 compounds have been subjected to extra precision (XP) docking using the default setting with the OPLS3 force field to generate a final in silico hit list of natural products. Following a docking score calculation, the compounds were prioritized and chosen for additional research depending on their abundance and commercial availability.

### Prediction of ADME and drug-likeness properties

The ADME properties of the active hits (liquiritin, oroxin B, ligustroflavone, and mulberroside A) were computationally predicted using Schrödinger’s QikProp module (version 2024-2) within the Maestro molecular modeling suite^9^. Small molecule structures were first prepared via geometric optimization in LigPrep with the OPLS4 force field, followed by enumeration of ionization states at physiological pH (7.0 ± 0.5) using Epik. Stereoisomers and tautomers were systematically generated through LigPrep’s conformational sampling protocol. QikProp calculations were performed to assess critical physicochemical and pharmacokinetic metrics, including molecular weight, partition coefficient, hydrogen bond donor and acceptor counts, Caco-2 permeability (QPPCaco), blood-brain barrier penetration (QPlogBB), and human oral absorption (%HOA). Drug-likeness was also evaluated using Lipinski’s Rule of Five and Jorgensen’s #stars metric, which quantifies deviations from 95% of known drug-like compounds. Validation of resulting hits also included benchmarking against 200 orally bioavailable DrugBank reference drugs, detection of reactive functional groups via QikProp’s 30 predefined alerts that could cause false positive results in hit discovery, and solubility prediction using the QPlogS model. Central nervous system activity was mapped using multiparameter optimization scores, and the risk of hERG channel inhibition was also evaluated as an important indicator of drug safety.

To complement the QikProp-based ADME-Tox assessment, we performed additional pharmacokinetic and drug-likeness predictions for the most promising compound, liquiritin, using the SwissADME web tool. SwissADME assesses essential physicochemical characteristics, such as molecular weight, lipophilicity, solubility, and bioavailability through a range of validated predictive models^10^.

### Molecular dynamics (MD) simulations

#### Protocol for MD simulations

Initially, retrospective induced-fit docking (IFD) was undertaken for top hits to confirm pose conservation versus rigid docking. IFD was performed using the Schrödinger software suite, which employs a protocol combining Glide docking and Prime structure refinement to account for protein flexibility and ligand-induced conformational changes during binding. Secondly, the MD simulations were undertaken using the Desmond simulation program of Schrödinger LLC^11^.

Protein-Inhibitor complexes were then prepared for MD simulations as shown in Sect. 2.1.2. In all runs, the NPT ensemble was employed with a 300 K temperature and a 1 bar pressure. The length of the simulation was 300 ns with a relaxation time of 1 ps. For all simulations, the OPLS3 force field parameters were used^12^. The cutoff radius in Coulomb interactions was set to 20.00Å. The boundaries of the orthorhombic periodic box were created 10 Å away from the protein atoms. Water molecules were clearly depicted using the transferable intermolecular potential applying the three points (TIP3P) model^13^. Using Desmond’s system builder tool, the salt concentration was set at 0.15 M NaCl to neutralize the system^14^. The Martyna − Tuckerman − Klein chain coupling system was employed for pressure control at 1 atm with a coupling constant of 2.0 ps, and applying the scheme of Nosé−Hoover chain coupling was used for temperature control at 303.15 K^15^. Non-bonded forces were calculated using a RESPA integrator, where the short-range forces were restructured at each step, and the long-range forces were updated every three steps. The trajectories were kept at 20 ns intervals for MD analysis. Using the simulation interaction diagram tool included in the Desmond MD package, the behavior and interactions between the studied ligands and the HER2 protein were investigated. Measurements of RMSD and root mean square fluctuation (RMSF) for the ligand and protein atom positions over time were used as trackers for the stability of MD simulations.

#### MD trajectory analysis and prime MM-GBSA calculations

The simulation interactions diagram panel of Maestro software was utilized to track the interaction functions reflecting the stability of studied ligand-protein complexes. The molecular mechanics generalized born/solvent accessibility (MM–GBSA) was accomplished to compute the free energies of ligand binding and the energies of ligand strain for docked compounds over the 300 ns period applying the thermal_mmgbsa.py python script provided by Schrodinger. This python script takes a Desmond trajectory file, divides it into individual snapshots, runs the MM-GBSA calculations on each frame, and outputs the average computed binding energy. The binding energy was estimated according to the following equation:

$$\:{\varDelta\:\text{G}}_{\text{b}\text{i}\text{n}\text{d}}={\text{E}}_{\left(\text{m}\text{i}\text{n}\text{i}\text{m}\text{i}\text{z}\text{e}\text{d}\:\text{c}\text{o}\text{m}\text{p}\text{l}\text{e}\text{x}\right)}-{\text{E}}_{\left(\text{m}\text{i}\text{n}\text{i}\text{m}\text{i}\text{z}\text{e}\text{d}\:\text{l}\text{i}\text{g}\text{a}\text{n}\text{d}\right)}-{\text{E}}_{\left(\text{m}\text{i}\text{n}\text{i}\text{m}\text{i}\text{z}\text{e}\text{d}\:\text{r}\text{e}\text{c}\text{e}\text{p}\text{t}\text{o}\text{r}\right)}$$

### In vitro assays

#### Biochemical HER2 kinase assay

The Z’-LYTE™ Kinase Assay-Tyr6 Peptide kit (Thermo Fischer Scientific™, Madison, WI, USA) was employed to evaluate the potential of selected natural products to suppress the catalytic activity of HER2 kinase. The assay adopts a Fluorescence Resonance Energy Transfer (FRET) based method in a cell-free coupled-enzyme format with no use of antibodies. The assay method mainly relies on the difference in sensitivity of phosphorylated and non-phosphorylated peptides to proteolytic cleavage. This method is a ratio metric method that calculates the Emission Ratio of donor emission divided by acceptor emission after excitation of the donor fluorophore at 400 nm. In this assay, 20 µL/well reactions containing kinase buffer, 200 µM ATP, 4µM Z’-LYTE Tyr6 Peptide substrate, 2500 ng/mL HER2 kinase, and drugs of interest functioning as inhibitors were set up in 96-well plates. Each well received 10 µL of site-specific protease development solution after one hour of room temperature incubation. The incubation continued for a full hour. Following process termination, the kinase inhibitory activity and/or peptide substrate cleavage status were assessed using a plate reader (BioTek FLx800™) to measure the ratio of fluorescence signal at 445 nm (coumarin)/520 nm (fluorescein).

The IC_50_ value for each natural product was computed using XLfit^®^ 4.1 software (ID Business Solutions Inc. (IDBS), Emeryville CA), applying non-linear regression of log concentration against the percentage of HER2 inhibition.

Oroxin B (CAT#SMB00340), liquiritin (CAT#L8045), delphin (CAT#PHL89626), mulberroside A (CAT#PHL80501), ligustroflavone (CAT#PHL83520) and positive controls as tyrphostin AG 1478 (CAT#T4182) and lapatinib (CAT#SML2259) were procured from Sigma-Aldrich (St. Gallen, Switzerland). Wild-type HER2 recombinant human protein was purchased from Thermo Fischer Scientific™ (CAT# PV3590).

#### Cell lines and culture conditions

The HER2-driven BT-474 and SKBR3 human breast cancer cell lines were grown in RPMI 1640 media containing 10% FBS and 2 mmol/L glutamine as supplements. To further avoid contamination, 100 U/mL of penicillin G and 100 µg/mL streptomycin were included. MCF10A cells were plated in DMEM/F12 supplied with 5% horse serum, 0.5 µg/mL hydrocortisone, 20 ng/mL EGF, 100 U/mL penicillin G, 100 ng/mL cholera toxin, 100 µg/mL streptomycin and 10 µg/mL insulin. All cell lines were subcultured according to standard protocols after becoming 80% confluent and maintained in a humidified incubator at 37 °C under 5% CO_2_. Liquiritin and oroxin B were initially dissolved in a volume of sterilized DMSO to prepare 10 mM stock solutions for all assays. The working solution of each test concentration was freshly prepared in the proper culture medium immediately before use. To prepare the vehicle-control (DMSO), the maximum volume of DMSO required to prepare the tested natural products was added to the suitable medium type. In this respect, the final DMSO concentration was kept constant throughout all treatment groups in each experiment and never went over 0.1%. Staurosporine and lapatinib were included in all experiments as positive controls, and their IC_50_ values were calculated for comparison.

#### Measurement of viable cell number

The 3-(4,5-dimethylthiazol-2yl)-2,5-diphenyl tetrazolium bromide (MTT) colorimetric test was used to estimate the viable cell count. The optical density of each sample was recorded at 450 nm using a microplate reader (ROBONIK P2000 Eia reader). The number of cells per well was determined using a hemocytometer against a standard curve prepared by plating different cell counts (1,000–60,000 cells per well) at the start of each experiment.

#### MTT proliferation assay

SKBR3 and BT-474 cells in exponential growth were seeded in 96-well culture plates at a density of 1 × 10^4^ cells per well (3 wells/group). Cells were allowed to adhere overnight in a humidified CO_2_ incubator at 37 °C while growing in FBS-containing RPMI-1640 media. Cells were then washed with PBS, divided into different groups, treated with 0.4, 1.6, 6.3, 25, and 100 µM doses of tested compounds or DMSO in serum-free media, and re-incubated for 48 h. It is worth noting that the treatment serum-free media was supplemented with 0.5% FBS to keep the cells viable during the experiment. Treatment media were then substituted with fresh ones and each well was also supplied with 50 µL of fresh MTT solution (1 mg/mL), and the plates were incubated for an additional four hours at 37 ºC. The color reaction was stopped by replacing the media in each well with 100 µL of DMSO, and incubation was resumed for 20 min to allow complete dissolution of the formed formazan crystals. Finally, absorbance was measured at λ 450 nm using a BIOLINE ELIZA plate microreader. Results were expressed as the mean percentage of viable cells as compared to the vehicle-treated control group. The % cell survival was determined according to the following equation: % survival = (Cell No. treatment/Cell No. DMSO) x 100.

For cytotoxicity assessment against the non-tumorigenic MCF10A mammary epithelial cell line, cells in exponential growth were seeded at a density of 1 × 10^4^ cells/well into 96-well plates (3 wells/group). The cells were allowed to adhere overnight in DMEM/F12 media containing 5% horse serum in a humidified CO_2_ incubator. The following day, cells were treated with varying doses of tested compounds, staurosporine, or vehicle in serum-free defined media after being washed with PBS, split into distinct treatment groups, and incubated for an additional 24 h at 37 °C under 5% CO_2_. Viable cell count was assessed using the MTT assay.

Selectivity index (SI) was calculated as the ratio of the IC_50_ of each compound in normal cell line (MCF10A) to IC_50_ in the corresponding cancer cell line (BT-474 or SKBR3) providing a quantitative measure of the differential cytotoxicity of the tested compounds.

#### Wound healing assay

In a sterile flat-bottom 24-well plate, SKBR3 and BT-474 breast cancer cells were plated at a density of 1 × 10^5^ cells/well. Cells were then maintained overnight in a humidified incubator at 37 °C under 5% CO_2_ until a confluent monolayer was attained in each well. Wounds were then inflicted across each cell monolayer using a sterile 200 µL pipette tip. Media was aspirated, and cells were washed twice with PBS to remove floating cells and cell debris. Fresh serum-free media was placed up against the well wall to cover the bottom of the well and prevent further cell detachment. Incubation of cells was resumed for five hours at 37 °C in a humidified CO_2_ incubator. Subsequently, the media were substituted with fresh ones containing various doses of liquiritin, oroxin B, or DMSO as a vehicle control (3 replicates/group) along with EGF (100 ng/mL) as a scattering factor. Wound closure was microscopically monitored in vehicle-treated control wells, and the time frame for wound closure was determined to be 72 h. Before the closure of wounds in the vehicle-treated control wells, the media were removed, cells were washed with PBS and completely fixed using cold methanol for 20 min at 4 °C. An inverted VistaVision microscope was used to take pictures of the wounds monitored at zero and 72 h with a VistaVision Still camera (VWR, Radnor, PA).

Results were expressed as a percentage of cell migration according to the following Equation^16^:

$$Percent~cell~migration = \frac{{{\text{T}}^{{\underset{\raise0.3em\hbox{$\smash{\scriptscriptstyle-}$}}{\text {o}} }} - {\text{T}}_{t} - {\text{T}}_{{DMSO}} ~}}{{{\text{T}}^{{\underset{\raise0.3em\hbox{$\smash{\scriptscriptstyle-}$}}{\text {o}} }} - {\text{T}}_{{DMSO}} ~}}~X~100$$

Where **T**_**t**_ is the wound thickness in treatment wells, **T**_**DMSO**_ is the thickness of the wound in control wells treated by DMSO, and **Tº** is the thickness of the wound at zero time. Wound thickness was calculated in three or more randomly selected fields per treatment group.

#### Transwell cell migration assay

The in vitro transwell migration assay was conducted using an HTS-96 transwell permeable support chamber with 8 μm pores (Corning, USA) equipped with polyvinylpyrrolidone-free polycarbonate (PVPF) filters. Separately, SKBR3 and BT-474 cells (0.5 × 10^5^) suspended in RPMI 1640 containing 10% fetal bovine serum and untreated or pretreated with liquiritin at concentrations equivalent to its respective IC_50_ values (determined via prior proliferation assays), were added to the upper compartment, and serum was added to the bottom compartment to induce cell migration. The plate was incubated for 24 h at 37 °C. Then, the transwell inserts were incubated in the receiver plate with Calcein AM/cell dissociation solution at 37 °C in 5% CO_2_ incubator for 1 h, followed by measuring the fluorescence using a fluorescence plate reader with 485 nm excitation and 520 nm emission filter package. The relative fluorescence units were converted to cell count using a standard curve. The number of cells that moved to the lower side of the filter was used to assess migratory characteristics. The experiment in both cell lines was conducted in triplicate and data are presented as the mean ± SD.

#### Analysis of cell cycle progression using flow cytometry

To evaluate liquiritin’s impact on cell cycle progression, a propidium iodide (PI)-based flow cytometry assay was performed using the Cell Cycle Analysis Kit (ab139418, Abcam) following the manufacturer’s protocol. BT-474 and SKBR3 cells were seeded into 100 mm culture plates at a density of 1 × 10^6^ cells/plate in RPMI 1640 medium supplemented with 10% FBS and incubated overnight. To synchronize cells in the G1 phase, cultures were rinsed twice with PBS and maintained in serum-reduced medium (0.5% FBS) for 48 h. Synchronized cells were subsequently treated with liquiritin at concentrations equivalent to its respective IC_50_ values (determined via prior proliferation assays) in serum-free media for 24 h.

Post-treatment, cells were detached using trypsin, washed with ice-cold PBS, and fixed in 66% ethanol at 4 °C for 2 h. Fixed cells were rehydrated in PBS and stained with a freshly prepared PI solution (1 mg/mL PI, 5,500 U/mL RNase A in PBS) for 30 min at 4 °C in the dark. DNA content was quantified using a BD FACS Calibur flow cytometer (BD Biosciences, San Jose, CA), with 10,000 events analyzed per sample. Cell cycle phase distributions (G0/G1, S, G2/M) were calculated from histogram data generated by Cell Quest software (BD Biosciences). All assays were performed in triplicate to ensure reproducibility.

#### Apoptosis detection via Annexin V staining

To evaluate the induction of apoptosis, the translocation of phosphatidylserine to the outer cell membrane, as a hallmark of early apoptosis, was detected by the binding of annexin V, using the Annexin V-FITC Early Apoptosis Detection Kit (BD BioVision). Cells were seeded at a density of 5 × 10^5^ cells per 100 mm culture plate and allowed to adhere overnight. Subsequently, cells were cultured for 48 h in serum-reduced medium (0.5% FBS) supplemented with either the control or liquiritin at doses equivalent to its respective IC_50_ values (determined via prior proliferation assays). At the end of the treatment period, cells were harvested by trypsinization, washed twice with ice-cold PBS, and resuspended in 500 µL of chilled 1X Annexin V Binding Buffer. To each sample, 5 µL of Annexin V-FITC conjugate and 5 µL of propidium iodide (PI, 50 µg/mL) were added. The cell suspensions were incubated for 5 minutes on ice in the dark to allow for staining.

Flow cytometric analysis was performed using excitation at 488 nm and emission at 530 nm for FITC detection (FL1 channel) and phycoerythrin emission settings for PI detection (FL2 channel). Data acquisition and analysis were facilitated by Cell Quest software (BD Biosciences), with dot plots divided into four quadrants to distinguish different cell populations: lower left (LL) for viable, non-apoptotic cells (negative for both annexin V and PI); lower right (LR) for early apoptotic cells (annexin V-positive, PI-negative); upper left (UL) for dead or necrotic cells (PI-positive, annexin V-negative); and upper right (UR) for late apoptotic cells (double-positive). All assays were conducted in triplicate to ensure reliability and reproducibility.

#### RNA extraction and real-time PCR for caspases’ gene expression investigation

RNA extraction was performed using Qiagen total RNA extraction kit from seeded BT-474 cells untreated and treated with liquiritin at a concentration equivalent to its respective IC_50_ value (determined via prior proliferation assays) according to the manufacturer’s manual. NanoDrop Lite Plus spectrophotometer™ (ThermoScientific, USA) was used for quantitation and purity evaluation of the extracted total RNA. cDNA synthesis and real-time quantitative PCR were performed in one reaction tube using iScript™ One-Step RT-PCR Kit with SYBR^®^ Green (Bio-Rad, USA) using primers listed in Table S2 per the instruction’s manual. The relative gene expression of caspases 3, 8, and 9 was analyzed using the 2^−ΔΔCT^ method along with untreated BT-474 cells as control and Glyceraldehyde-3-phosphate dehydrogenase (GAPDH) as a housekeeping gene. The data were expressed as fold change in gene expression.

#### Western blot analysis

SKBR3 and BT-474 were plated in RPMI-1640 media, washed, and incubated for 72 h. They were lysed in cold lysis buffer, frozen, collected, sonicated, and centrifuged for 10 min under cooling. The stacking and resolving solutions for polyacrylamide gels were prepared using various solutions, including 30% acrylamide, 0.5 M Tris, 10% SDS, 10% Ammonium persulfate, and 0.005 mL TEMED in 3.4 mL water and 3.3 mL water respectively. The ReadyPrepTM protein extraction kit was used to extract total protein from samples. Protein concentration was determined using the Bradford Protein Assay Kit. Samples were mixed with Laemmli sample buffer, pH adjusted and boiled for five minutes at 95 °C before loading onto SDS-polyacrylamide gels. Proteins in each sample were mixed, boiled, and loaded onto SDS-polyacrylamide gels. They were separated using the SDS-PAGE method, using the TGX Stain-Free™ FastCast™ Acrylamide Kit and a Cleaver electrophoresis unit. Proteins were transferred from gels to PVDF membranes using transfer sandwiches. Filter paper, membrane, gel, and filter paper were stacked in a sandwich. Gels were blotted onto PVDF membranes via electrophoresis using a Bio-Rad Semi-dry Electroblotter for 30 min. PVDF membranes were blocked with 5% non-fat dry milk in TBST buffer for 2 h at room temperature to minimize non-specific protein interactions with the antibody. This study utilized primary antibodies specific to p-HER2 or t-HER2 proteins, prepared in TBST at a 1:1,000 dilution and incubated overnight at 4 °C. Membranes were washed three times with TBST to remove excess primary antibodies, diluted with secondary antibodies, and incubated with horseradish peroxidase-linked anti-rabbit IgG secondary antibody solutions. Membranes were rinsed three times. The study used Western ECL™ substrate to visualize blots on X-ray film, incubating membranes with ECL solutions A and B. Protein bands were acquired using a CCD camera-based system, with β-actin as a housekeeping protein. The intensity of each band was quantitated using ImageLab.

### Kinase profiling

Liquiritin was screened at 0.1 µM dose against a panel of 15 human kinases using SelectScreen^®^ kinase profiling services offered by ThermoFisher Scientific™ (Madison, WI, USA). The percent inhibition of Liquiritin was assessed against each kinase in an automated high-throughput screening system using the time-resolved fluorescence resonance energy transfer Z´-LYTE™ kinase assay. Technology overview, protocols, and assay conditions are accessible at https://x.gd/UgBBB.

### Statistics

The results are shown as means ± SD of at least three independent experiments. Differences among various treatment groups were evaluated applying the analysis of variance (ANOVA) followed by Dunnett’s test using PASW statistics version 18. A difference of *P* < 0.05 was regarded statistically significant with reference to vehicle-treated control groups. The IC_50_ values were computed applying non-linear regression curve fit analysis using GraphPad Prism software version 5.01 (GraphPad Software, CA).

## Results and discussion

### Molecular docking validation and structure-based virtual screening

The study used a docking protocol to ensure precise ligand binding to the active site, with a low root mean square deviation. A test run was conducted using 18 established HER2 kinase inhibitors (Table S1), including FDA-approved lapatinib, tucatinib, and neratinib, in addition to 1000 decoys embedded at the Schrodinger suite to validate the docking procedure. Furthermore, the accuracy of GLIDE docking in predicting binding poses and docking scores of protein-ligand complexes was evaluated. A ROC plot was generated, and multiple predictive power metrics were computed. The docking algorithm demonstrated high sensitivity in identifying all standard HER2 inhibitors and good specificity in distinguishing actives from decoys. Pivotally, the adopted docking protocol was able to fish active ligands from a seeded random set once the top 2%, 5%, and 10% of the total set were taken into consideration. The maximum attainable enrichment factor values EF (2%), EF (5%), and EF (10%) of the docking approach are 52, 20 and 10, respectively (Table S3).

The docking protocol BEDROC showed an optimal score of 1 for early detection of actives from decoys and outstanding performance in ranking actives at the top of the hit list. Lapatinib, epertinib, and allitinib were the top hits, with docking scores of -12.0, -11.4, and − 10.9 kcal/mol, respectively (Table S4). Compounds maintained hydrogen bonds with Met 801 at a distance of 2.8 Å. Both lapatinib and epertinib adopted a perfect U-shaped conformation and formed an extra hydrogen bond with Asp 808 and a salt bridge with its side chain, indicating their superior binding affinity over allitinib (Fig. S1). Epertinib showed a unique interaction with Phe 864 in the DFG motif, indicating the accuracy, specificity, and validity of the docking protocol, prompting a structure-based virtual screening campaign.

Structure-based virtual screening was performed for the compiled natural products library (present in the supplementary files) against HER2 kinase using Schrodinger software, with a maximum of 10 poses per natural product. In an attempt to enhance the quality of the obtained in silico predictions, the virtual screening campaign has been designed to consist of three successive stages of molecular docking in an ascending order of precision, starting with HTVS, then moving to SP protocol, and finally reaching an extra-precision XP protocol. After the initial HTVS stage, only 541,589 were successfully fitted within the active site of the HER2 kinase and were ranked according to their corresponding docking scores. In the majority of virtual screening campaigns, some of the top-ranked hits will be selected for biological validation using in vitro assays. However, this approach was not adopted in this study to minimize the possibility of false-positive hits that may arise due to the inaccuracy of existing scoring functions in discriminating poor from suitable binders^17^. Instead, out of the 541,589 hits, the top-ranked 10,000 poses comprising 8268 natural products were passed out to a second round of molecular docking, applying standard precision (SP) protocol. As a final refining step of the in silico ranking predictions, the top 500 poses representing 459 natural products from the SP stage were subjected to a third cycle of molecular docking, implementing the extra precision (XP) protocol (Scheme S1).

Table 1 displays the top forty hits along with their corresponding docking scores, which showed the highest binding affinity when docked within the HER2 kinase domain (PDB: 3RCD). Among the top-ranked 40 hits, five hits were selected (delphin, ligustroflavone, oroxin B, liquiritin, and mulberroside A) for in vitro testing. Further investigation of the natural sources of these 40 hits guided the choice of the five hits, representing a range of docking scores between − 13.5 and − 10, thereby capturing both high- and moderate-affinity candidates. The selection was further guided by previous reports of anticancer activity of their natural sources, abundance and accessibility of the natural sources, possibility of compound extraction in high yield, and commercial availability to ensure feasibility for future development. This multifaceted approach ensured that our validation set was not only computationally promising but also biologically relevant and practically feasible for further development.

**Table 1:** List of top 40 hits with HER2 tyrosine kinase domain (PDB ID: 3RCD) ranked with respective docking scores.

#	Compound name	Docking score (kcal/mol)	Chemical class	Natural source	Ref.
1	Pseudohexafuhalol B	−20.59	Phlorotannin	*Brown algae (Sargassum spinuligerum)*	^18^
2	Hexafuhalol B	−18.08	*Phlorotannin*	*Brown algae* *(Carpophyllum angustifolium)*	^19^
3	Pseudopentafuhalol C	−16.96	*Phlorotannin*	*Brown algae* *(S. spinuligerum)*	^18^
4	Narirutin 4’-glucoside	−15.93	Flavonoid glycosides	*Citrus maxima*	^20^
5	Luteolin 7-Sophorotrioside	−15.36	Flavonoid glycosides	*Leptostomum macrocarpum*	^21^
6	Kaempferol 3-Rhamnosyl-(12)-[Xylosyl-(13)-Rhamnosyl-(16)-Galactoside]	−15.1	Flavonoid glycosides	*Astragalus caprinus*	^22^
7	Aceroside VIII	−14.55	Linear diarylheptanoids	*Betula platyphlla*	^23^
8	Swertiajaponin3’-*O*-Gentiobioside	−14.16	Flavonoid glycosides	*Phragmites australis*	^24^
9	6-Hydroxykaempferol 6-Methyl Ether 3-(6’’-*P*-Coumaroylglucoside)	−13.54	Flavonoid coumaroyl glycosides	*Paepalanthus species*	^25^
10	2-(4-Hydroxyphenyl)-Ethyl-(6-*O*-Caffeoyl)-Beta-D-Glucopyranoside	−13.45	Coumaric acids derivatives	*Fraxinus ornus*	^26^
11	Delphin (Delphinidin-3,5-*O*-diglucoside)	−13.37	Anthocyanidin glycosides	*Punica granatum*	^27^
12	Ligustroflavone (Nuezhenoside)	−12.99	Flavonoid glycosides	*ligustrum vulgare*, *L. lucidum*	^28^
13	Apigenin 7,4’-Diglucoside	−12.89	Flavonoid glycosides	*Salvia uliginosa*	^29^
14	Kaempferol 3-[6’’-(3-Hydroxy-3-Methylglutaryl) Glucoside]	−12.82	Flavonoid glycosides	*Citrus aurantifolia*	^30^
15	Kaempferol 7-Methyl Ether 3-Xylosyl-(12)-[Rhamnosyl-(16)-Glucoside]	−12.8	Flavonoid glycosides	*Cestrum nocturnum*	^31^
16	Kaempferide 3-*O*-neohesperidoside	−12.73	Flavonoid glycosides	*Costus spicatus*	^32^
17	Quercetin 7-Glucuronide	−12.67	Flavonoid glucuronides	*Verbascum lychnitis*	^33^
18	Kaempferol-3-*O*-robinobioside	−12.63	Flavonoid glycosides	*Camellia sinensis*	^34^
19	Teucrioside	−12.6	phenylpropanoid glycoside	*Teucrium chamaedrys*	^35^
20	Gossypetin 7,4’-Dimethyl Ether 8-Glucoside	−12.56	Flavonoid glycosides	*Zanthoxylum acanthopodium*	^36^
21	Quercetin7-Glucoside/ Quercimeritrin	−12.35	Flavonoid glycosides	*Cudrania tricuspidata*	^37^
22	Vicenin-2 (Apigenin 6,8-di-C-glucoside)	−12.27	Flavonoid glycosides	*Citrus* *Aurantium*,* C. aurantifolia*	^38^
23	Hypolaetin 7-*O*-Glucuronide	−12.23	Flavonoid glucuronides	*Stachys species*	^39^
24	Dihydrophelloside	−12.18	Flavonoid glycosides	*Phellodendron sachalinense*, *P. amurense*	^40^
25	7-*O*-Methyl morroniside	−12.06	O-glycosyl compounds	*Cornus officinalis*	^41^
26	Calyxin E	−11.83	Linear diarylheptanoids	*Alpinia blepharocalyx*	^42^
27	Oroxin B	−11.76	Flavonoid glycosides	*Oroxylum indicum*	^43^
28	Senburiside Iv	−11.69	Tannins	*Swertia franchetiana*	^44^
29	Hymenoside F	−11.65	Fatty acyl glycosides	*Hymenophyllum barbatum*	^45^
30	Tricetin 6-C-Glucoside-8-C-Arabinoside	−11.56	Flavonoid glycosides	*Plagiochila jamesonii*	^46^
31	Delphinidin 3-(6-Malonylglucoside)-5-Glucoside	−11.49	Anthocyanidin glycosides	*Cichorium intybus*	^47^
32	Pseudospumigin A	−11.4	Peptides	*Nostoc sp.*	^48^
33	Acacetin 7-Apiosyl (16)-Glucopyranoside	−11.39	Flavonoid glycosides	*Carthamus tinctorius*	^49^
34	Luteolin-5-*O*-Glucoside	−11.36	Flavonoid glycosides	*Cirsium maackii*	^50^
35	[(+)-Androyol]	−11.36	2-arylbenzofuran flavonoids	*Alluaudia dumosa*	^51^
36	Kaempferol 3-(2’’-*P*-Coumaryl-Alpha-L-Arabinopyranoside)	−11.33	Flavonoid glycosides	*Prunus spinosa*	^52^
37	Liquiritin	−11.32	Flavonoid glycosides	*Glycyrrhiza sp.*	^53^
38	Malvin Chloride	−11.24	Anthocyanidin glycosides	*Vitis amurensis*	^54^
39	Alatanin C	−11.22	Flavonoid glycosides	*Dioscorea alata*	^55^
40	Mulberroside A	−10.43	Stilbene glycosides	*Morus alba*	^56^

### Biochemical HER2 kinase activity assay (primary screen)

Utilizing the Z’-LYTE™ Kinase Assay-Tyr6 Peptide kit as a cell-free system for evaluating the inhibitory activity of HER2, the five natural products were evaluated for their capacity to inhibit HER2 phosphorylation. Since its kinase catalysis is necessary for all HER2-mediated activities, we attempted at this initial stage to validate the HER2 inhibitory ability of the five in silico hits directly on the HER2 purified kinase domain (amino acids 676–1255), that was in vitro phosphorylated to attain the maximum level of intrinsic kinase activity. In this assay, the substrate was Z’-LYTE™ Tyr6 peptide; thus, the changes in its phosphorylation can directly correlate with the HER2 kinase activity. In the meantime, tyrphostin AG 1478 was used as a positive control to ensure the validity of the assay. Lapatinib (Tykerb^®^) was included as a second positive control to benchmark virtual hits’ performance against clinically approved standards.

**Fig. 1:**
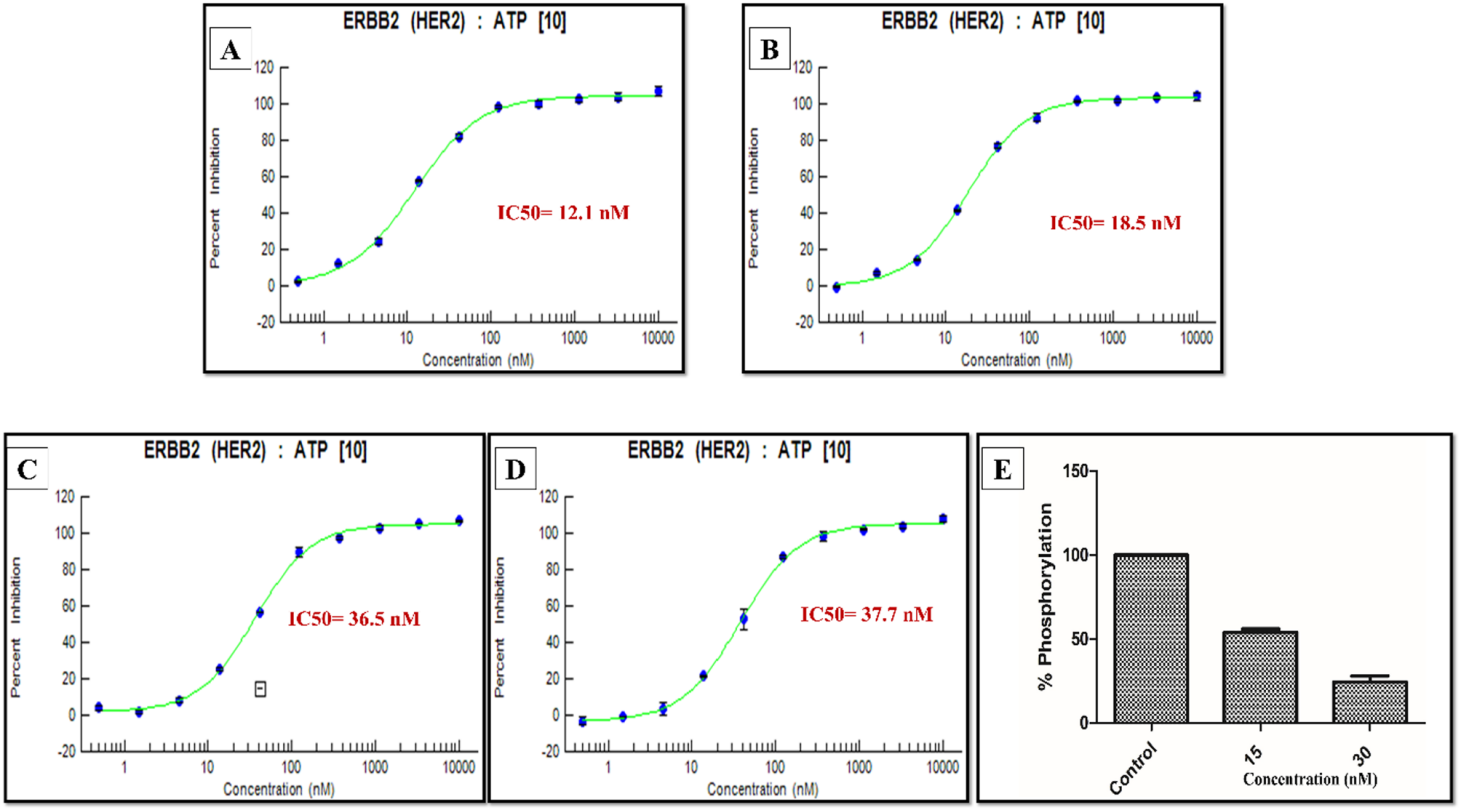
In vitro effect of the potential in silico hits on the phosphorylation of HER2 kinase at different concentrations. (**A**) Oroxin B, (**B**) Liquiritin, (**C**) Ligustroflavone, (**D**) Mulberroside A and (**E**) Lapatinib, using Z’-LYTE™ assay kit. Error bars represent the SD.

Interestingly, oroxin B, liquiritin, ligustroflavone, and mulberroside A significantly inhibited ATP-induced HER2 phosphorylation in a dose-responsive manner, with IC_50_ values of 12.1, 18.5, 36.5, and 37.7 nM, respectively (Fig. 1). However, there was no discernible suppression of HER2 phosphorylation by delphin. Notably, these values compare favorably to lapatinib, which achieved 54% inhibition at 15 nM and 75% inhibition at 30 nM in the same assay (Fig. 1E), suggesting an efficient and clinically relevant engagement with HER2 kinase domain. Importantly, the low nanomolar affinity exceeds the typical potency range of virtual-screening hits against cancer targets (1–25 µM), highlighting the exceptional performance of our computational approach relative to industry benchmarks and the validity of revisiting the natural chemical space when searching for new drug leads.

### Binding mode analysis and structure-activity relationship (SAR)

Following that, in an attempt to identify the structural determinants of HER2 inhibitory activity for the tested natural products, their predicted binding poses were examined using the Maestro interface of Schrödinger (Fig. S2). Studying these poses not only peered into potential binding patterns of investigated natural products inside the catalytic region of unphosphorylated HER2 but also helped to gain some preliminary structure-activity relationship insights for effective HER2 kinase inhibition. To do so, the predicted binding poses of studied natural products were correlated with their corresponding activity level in the biochemical assay as a primary screen (Fig. 1).

A detailed examination of the binding poses revealed some common essential features of HER2 binding affinity. Firstly, the existence of the carbonyl group in the pyran ring is crucial for activity due to its involvement in an essential single-point hydrogen bonding interaction with the backbone of Met 801 at the hinge region (Fig. S2A,C,E). This pattern was consistent among the three active flavonoid glycosides: oroxin B, liquiritin, and ligustroflavone, which showed HER2 potency in the low nanomolar range (Fig. 1). Alternatively, delphin, which lacks the pyran’s carbonyl group, failed to fulfill such essential hydrogen bonding interaction within the hinge region and consequently exhibited poor activity in the HER2 biochemical experiment (Fig. S2B). Conversely, mulberroside A retained its activity even though it lacked such an essential pharmacophoric group since it is a stilbene and not a flavonoid. This activity can be clarified by the ability of mulberroside A to exert an alternative hydrogen bond through its glucosyl hydroxyl group and another critical amino acid, ASP 863 (Fig. S2D). Met 801 and Asp 863 are among the critical amino acids that determine whether a small molecule is active or not.

Secondly, docking simulations suggested that the best possible scenario for HER2 binding affinity is the engagement of one pharmacophore to Met 801 in the hinge from one end and a second pharmacophore to Asp 808 from the other, all while maintaining a spacer (linker of 8–9 atoms) (Fig. S2). This typically matches the case of oroxin B, which demonstrated the highest activity in the cell-free assay (Fig. 1). Consistently, the activity started to decrease as the spacer exceeded the proposed value, as seen in the cases of liquiritin (12 atoms), ligustroflavone (15 atoms), and mulberroside A (18 atoms). This linker hypothesis could also explain why ligustroflavone and mulberroside A have similar activity levels even though their scaffolds are quite different.

Thirdly, docking simulations predicted that the binding affinity of flavonoid glycosides within the HER2 kinase pocket is influenced by the number, type, and arrangement of sugars at positions 3,5 and 7 of the flavonoid scaffold. It is highly preferred that there would be two glucose moieties at position 7. Increasing the number of sugars at this position or introducing other types of sugars, such as rhamnose instead of glucose, had a negative impact on the activity for two main reasons. The pocket size will not be sufficient to accommodate the increased bulkiness in this case; additionally, rhamnoses are relatively less hydrophilic than glucose, and since they will be solvent-exposed, this would create an energy penalty and negatively impact the binding affinity. Ligustroflavone has, therefore, demonstrated less activity in this circumstance. In the same context, having only one sugar unit at position 5 while shifting the other sugar unit to position 3 resulted in a complete loss of HER2 kinase inhibition, as seen in the case of delphin (Fig. S2B).

Meanwhile, the docking studies suggested that ring B should be oriented to the solvent near the open end of the binding site for optimal results, as in the case of oroxin B (Fig. S2A), docking simulations showed that if the sugar position is altered to position 4’, the compound will be inversely docked with ring A protruding towards the solvent, and the activity will relatively decrease due to missing an essential interaction with Asp 808. Therefore, such pose is not preferred over oroxin B’s conformation (Fig. S2C). These docking results imply that a hydroxylated version of oroxin B, introducing one or more polar groups to ring B, would have greater activity than unsubstituted oroxin B (Fig. S2A).

Docking simulations also predicted some favorable features that would have a slight impact on HER2 binding affinity, including the presence of a double bond between positions 2 and 3 of the flavonoid scaffold and a free hydroxyl group at position 5. In the case of liquiritin, the lack of a double bond between C2 and C3 may contribute to its decreased activity since, in contrast to oroxin B, it provided the scaffold with more undesired flexibility, which may also account for its inverted docking position within the HER2 kinase domain. Conversely, the presence of the free hydroxyl group at position 5, as demonstrated by ligustroflavone, is favorable since it provides an additional binding pharmacophore to engage with MET 801 in the hinge region (Fig. S2A,E).

**Fig. 2:**
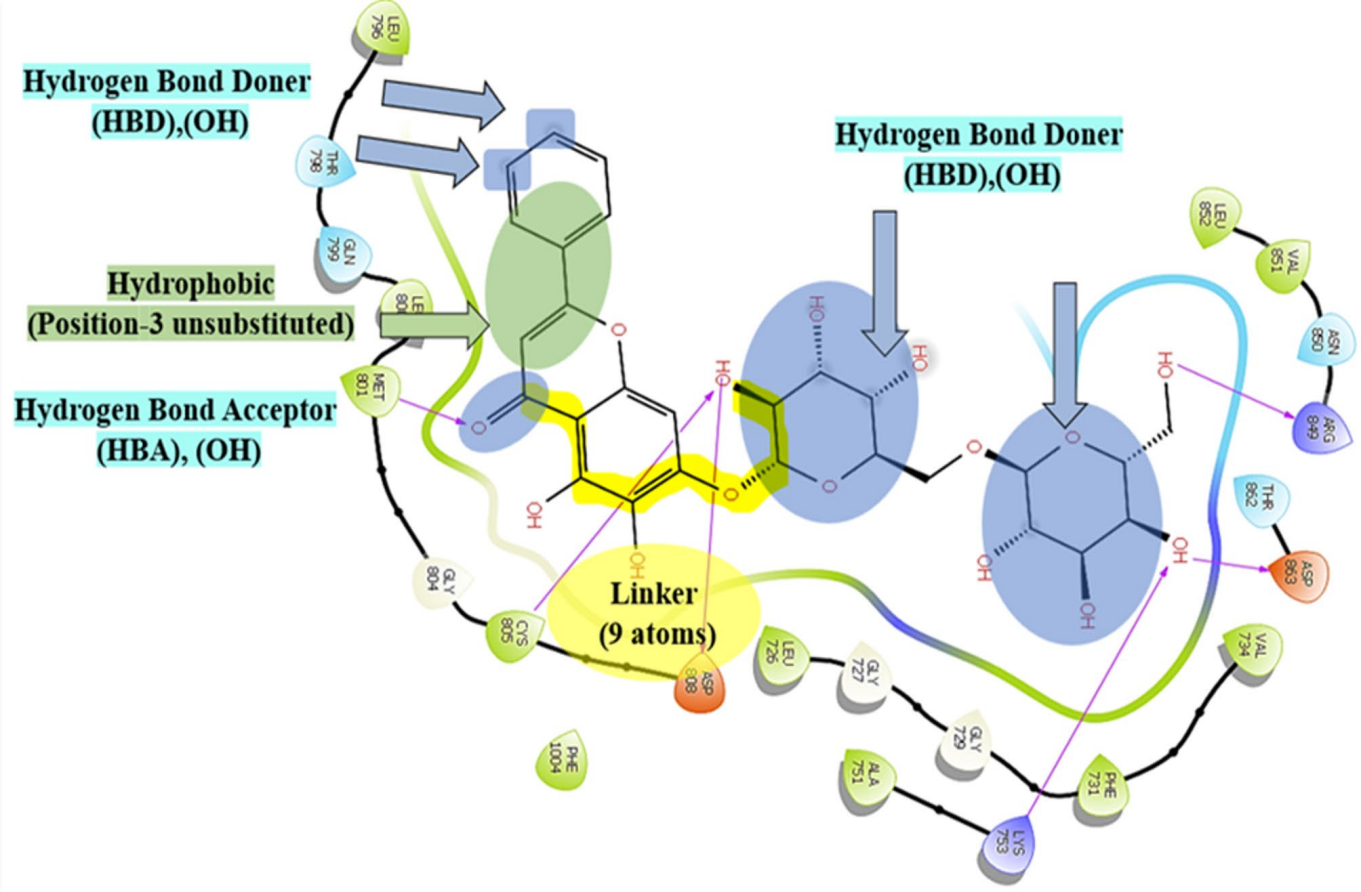
Predicted structure-activity relationship (SAR) of the discovered natural products for effective HER2 inhibition at the biochemical level.

Taken together, binding mode analyses and primary biochemical screening results provided some preliminary SAR insights for effective HER2 inhibition. The results highlighted the importance of the pyran’s carbonyl group and the optimal number of linker atoms, as well as the type, arrangement, and number of sugar units for orienting binding pharmacophores towards critical amino acids within the binding site. These predictions might illustrate, at least in part, the nanomolar activity level of oroxin B in the Z’-LYTE™ kinase biochemical experiment (Fig. 1). It also opened future directions of its optimization through ring B hydroxylation. Figure 2 compiles the preliminary SAR insights that have been concluded in this study.

### In silico ADME profiling and drug-likeness evaluation

ADME assessment is crucial for drug discovery, as it enables early evaluation of a drug’s physicochemical properties, safety profile, and pharmacokinetic behavior, helping to identify promising candidates and minimize late-stage failure. Following the primary screening run, we utilized Qikprop in Maestro to predict the ADME and drug-likeness attributes of the four active hits as shown in Table S5.

Among the virtual screening-derived HER2 inhibitors, liquiritin exhibited the most favorable ADME profile compared to oroxin B, ligustroflavone, and mulberroside A. While all compounds fell within the recommended molecular weight range of 130.0 to 725.0, liquiritin uniquely adhered to Lipinski’s Rule of Five (0 violations vs. 3 violations for others), with optimal LogP (-0.25 vs. -2.17 to -2.71), suggesting superior oral bioavailability. Notably, liquiritin demonstrated 50% predicted human oral absorption (medium qualifier), outperforming other hits (0% absorption) and aligning with natural product-derived drugs like quercetin glycosides^57^. QPPCaco, a model for the gut-blood barrier, predicts a drug’s intestinal wall penetration capacity in nanometers per second, crucial for oral absorption and bioavailability^58^. Liquiritn’s Caco-2 permeability (23 nm/s) approached the threshold for acceptable intestinal absorption (< 25 nm/s = poor), suggesting potential for formulation-enhanced bioavailability, whereas oroxin B, ligustroflavone, and mulberroside A exhibited negligible permeability (< 2 nm/s).

Drug pharmacokinetics and pharmacodynamics are impacted by plasma protein binding, which may lessen the efficacy of therapeutics. The admissible range for binding to human serum albumin, QPlogKhsa, is between − 1.5 and 1.5^59^. With values of -1.415 and − 0.723 for oroxin B and liquiritin, respectively, these two compounds were found to be within the ideal range (Table S5). This equilibrium between bound and free (active) drug forms helps to sustain therapeutic concentrations in the bloodstream, potentially enhancing the candidates’ suitability for clinical use, reducing the required dosing frequency, and improving overall efficacy^60^.

Critically, liquiritin showed a low HERG inhibition risk (log IC_50_ = -5), indicating a favorable cardiac safety profile comparable to lapatinib (log IC_50_ ≈ -4.8). While its blood-brain barrier penetration (QPlogBB = − 2.82) is limited, this minimizes off-target CNS effects, an advantage for peripheral HER2-targeted therapy. With 7 primary metabolites generated mainly through alcohol oxidation and aromatic hydroxylation, all typical of flavonoid Phase I metabolism, liquiritin demonstrated reduced metabolic complexity and lower risk of unexpected toxicity compared to the 9–10 metabolites observed for the other hits.

All compounds exhibited 2 violations of Jorgensen’s Rule of Three (maximum allowed = 3), with violations primarily linked to QPPCaco and number of primary metabolites, parameters common to flavonoid glycosides. While these deviations suggest potential formulation challenges, the violations of liquiritin in particular remained within acceptable limits for early-stage hits, supporting its prioritization for lead optimization. To maximize confidence in our ADME-Tox predictions, we chose to analyze our lead compound, liquiritin, using more than one reputable in silico tool. Alongside QikProp, we applied the SwissADME platform to assess its drug-likeness and pharmacokinetic properties. The outcomes from SwissADME (Table S6), covering crucial parameters such as molecular weight, lipophilicity, and bioavailability, were found to be in close agreement with those from QikProp. Specifically, liquiritin showed a molecular weight of 418.39 g/mol, a moderate lipophilicity (consensus log P around 0.25–1.46 depending on the model), and a bioavailability score of 0.55, indicating a reasonable probability of oral absorption. This alignment between results from distinct computational methods not only affirms the robustness of our predictions but also highlights the importance of cross-verification in computational drug discovery.

These attributes position liquiritin as a tractable lead for further optimization with balanced solubility, permeability and safety, key for advancing natural scaffolds into preclinical development. Its compliance with drug-likeness guidelines, coupled with moderate oral absorption, underscores its potential as a backbone for optimizing HER2-targeted therapeutics with improved bioavailability over synthetic kinase inhibitors.

Overall, liquiritin exhibited the most favorable ADMET profile, followed by oroxin B despite its oral bioavailability concern (Table S5). In contrast, mulberroside A and ligustroflavone displayed the poorest ADMET properties, violating 11 and 13 key drug-like parameters, respectively, and exceeding the majority of critical thresholds. Consequently, these compounds were excluded from further analysis.

### Molecular dynamics simulation

Based on the primary screen assay, docking results, and ADME predictions, oroxin B and liquiritin stood out to be the most promising natural products as HER2 inhibitors among the tested compounds. Initial IFD analysis of liquiritin and oroxin B revealed binding poses consistent with rigid docking results (Fig. S2). This conformational alignment indicated minimal ligand-induced receptor flexibility for these compounds, supporting the validity of our initial screening approach. Following that, oroxin B and liquiritin complexes with HER2 kinase domain protein were subjected to 300 ns MD simulations to verify their excellent docking scores and study their behavior within the active site of HER2 kinase.

#### RMSD and RMSF analyses

**Fig. 3:**
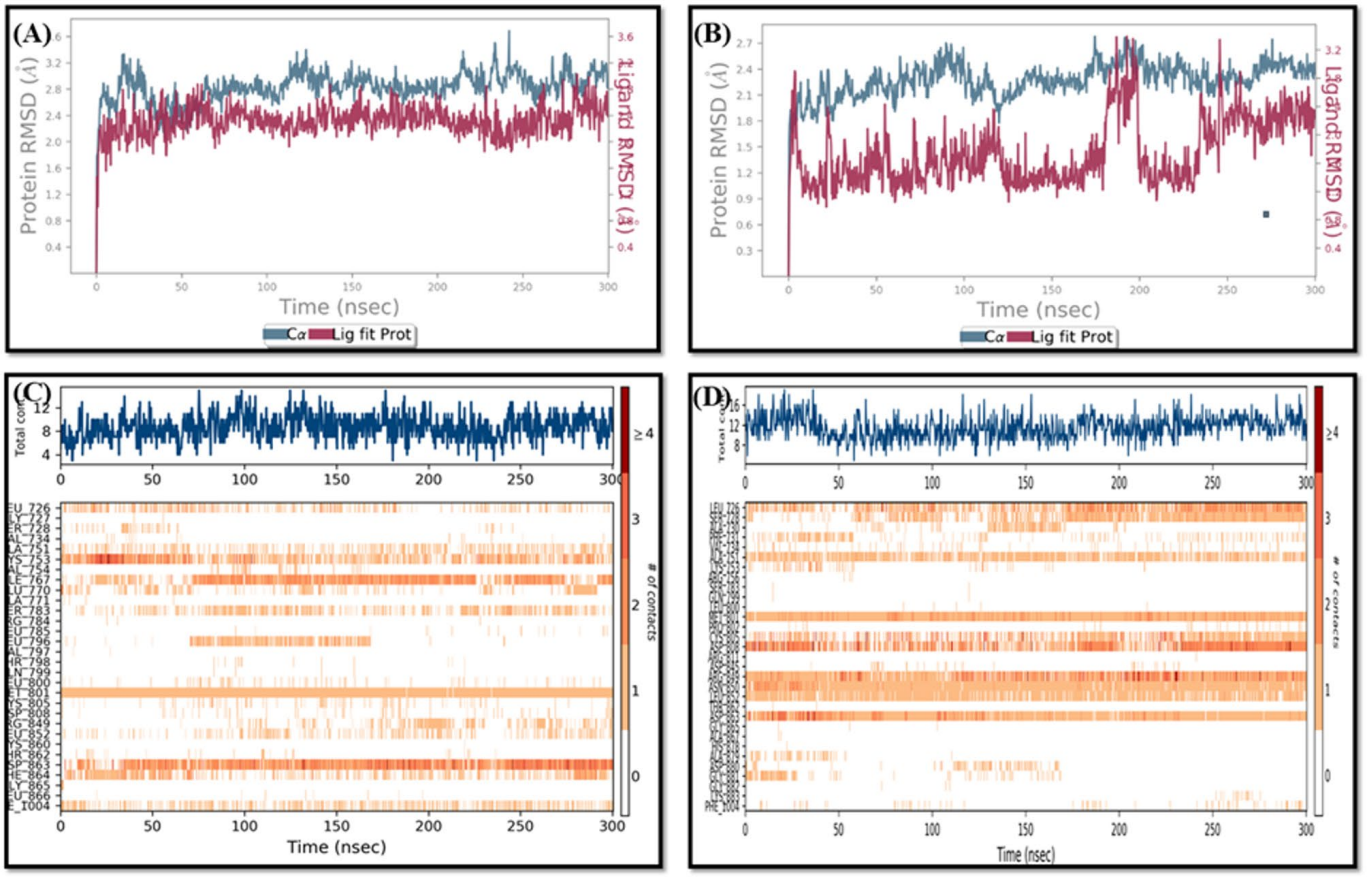
The ligands’ (right) and the complex’s (left) RMSDs as a function of simulation time for (**A**) Liquiritin and (**B**) Oroxin B and Heat map showing the total number of HER2–ligand contacts during the simulation time of 300 ns for (**C**) Liquiritin and (**D**) Oroxin B.

The oroxin B-HER2 and liquiritin-HER2 complexes’ Cα RMSD were monitored and plotted as a function of simulation time with respect to their initial position, (Fig. 3A and B). As can be seen in Fig. 3A and B, both complexes showed RMSD of less than 3.00 Å, which falls within acceptable limits. Such RMSD indicates that neither ligand affected the stability of the HER-2 conformation.

Next, The RMSD of the ligands was also monitored and plotted as a function of simulation time, (Fig. 3A,B). Oroxin B’s RMSD stabilized at 1.60 Å, rose transiently to 3.20 Å, then returned and finally increased to a steady 2.40 Å by the end of the simulation. Liquiritin’s RMSD started at 3.50 Å, gradually decreased, and stabilized at 2.00 Å by 180 ns until the end of the simulation, (Fig. 3A).

The RMSF measurements of the HER2 protein complexes with liquiritin and oroxin B are shown in Fig. S3. Except for the HER2 protein’s N- and C-terminals, RMSF values varied below 3.0 Å for both complexes, suggesting remarkable stability as a result of its improved stiffness. The only notable variation was observed between residues 160 and 180, where a loop lacking a clear structure was anticipated.

#### Binding interactions histogram and heat map analysis

The histogram of binding interactions for each investigated protein-ligand complex during the simulation time of 300 ns is shown in Fig. S4. Oroxin B-HER2 complex exhibited strong hydrogen bonding interactions with amino acids Met 801, Asp 808, Arg 849, Asn 850, and Asp 863 for more than 70% of the simulation time. It maintained critical hydrogen bonds with Met 801 and Asp 863 in the DFG motif. Oroxin B was able to form water-bridged hydrogen bonds with Leu 726, Cys 805, Asp 808, and Arg 849 (Fig. S4A). Liquiritin interacted with HER2 kinase through strong hydrogen bonds towards Met 801, Asn 850, Ala 730, and Asp 863, along with water-bridged hydrogen bonds toward residues Gln 799, Asp 845, Arg 849, and Asp 863. It also exhibited moderate hydrophobic interactions with Ala 731 and Phe 1004, with no ionic interactions observed within the HER2 kinase (Fig. S4B). Figure 3C and D-top panel display a timeline of liquiritin and oroxin B’s interactions with HER2 protein respectively, showing that both compounds maintained 8–12 specific contacts with HER2 protein. Heat maps revealed key HER2 amino acids mediating binding affinity for oroxin B and liquiritin. Oroxin B’s critical residues include Leu 726, Ala 751, Met 801, Asp 808, Arg 849, Asn 850, Leu 852, and Asp 863 (Fig. 3D-bottom panel). Liquiritin primarily anchored to the HER2 kinase via Lys 753, Met 801, Asp 863, and to a lesser extent through Leu 726, Ala 751, Ile 767, Ser 783, Phe 864, and Phe 1004 (Fig. 3C-bottom panel).

It is worth noting that oroxin B and liquiritin maintained specific contacts with their anchoring residues in most simulation trajectory frames, indicating their potential as effective HER2 kinase inhibitors. They also maintained strong crucial interactions with the hinge and DFG motif of HER2 kinase. Furthermore, these results corroborated previous findings showing the necessity of Met 801 and Asp 863 residues within the HER2 protein’s binding region for interactions with putative inhibitors^61^.

#### Ligand properties study analysis

Analysis of key ligand properties, including Molecular Surface Area (MolSA), Solvent Accessible Surface Area (SASA), Polar Surface Area (PSA), intramolecular Hydrogen Bonds (intraHB), Radius of Gyration (rGyr), and Root Mean Square Deviation (RMSD), provides insight into the binding characteristics of oroxin B and liquiritin with HER2 (Fig. S5). For the oroxin B-HER2 complex, RMSD values equilibrated around 1.2 Å, indicating consistent ligand conformation, while rGyr remained low and stable, reflecting a compact and stable protein-ligand interaction (Fig. S5A-i and ii). Oroxin B also maintained a stable internal hydrogen bond and minimal fluctuations in MolSA (~ 435–465 Å², equilibrating at 450 Å²), with SASA and PSA stabilizing at approximately 120 Å² after initial fluctuations (Fig. S5A-iii-vi).

Similarly, the liquiritin-HER2 complex exhibited negligible RMSD variation, with a stable equilibrium rGyr of 5.30 Å, and strong intramolecular hydrogen bonds, suggesting good compactness and conformational stability. Liquiritin’s MolSA ranged narrowly (464–488 Å², equilibrating at 475 Å²), while SASA and PSA showed lower fluctuations compared to oroxin B (Fig. S5B). Together, these findings demonstrate that both ligands form stable complexes with HER2, with oroxin B demonstrating a slightly more rigid binding mode compared to liquiritin throughout the simulation.

#### MD trajectory analysis and prime MM-GBSA calculations

To rigorously assess time-dependent binding free energy changes during MD simulations, we calculated MM-GBSA energies for oroxin B and liquiritin within HER2 kinase, a method superior to empirical scoring functions^62^. Using Schrödinger’s thermal_mmgbsa.py script, the study calculated average binding free energies for oroxin B and liquiritin to HER2 kinase as − 51.41 ± 3.64 kcal/mol and − 63.70 ± 6.21 kcal/mol, respectively (Table S7). Accordingly, both HER2 complexes are comparably stable with more preference for liquiritin. Interestingly, these results placed liquiritin ahead of oroxin B as a putative HER2 inhibitor despite the better rank of the latter in rigid docking studies.

### Biological characterization of the potential in silico hits

#### Cell proliferation MTT assay (counter screen)

Based on their promising biochemical data, oroxin B, liquiritin, ligustroflavone, and mulberroside A were further assessed in additional cell-based functional assays to validate the docking and cell-free results in a cellular context. The MTT cell proliferation assay was primarily chosen as a counter screen to evaluate the ability of the four hits to inhibit the growth of two HER2-driven human breast cancer cell lines, namely SKBR3 and BT-474 cells. In this counter screen, five different doses (0.4, 1.6, 6.3, 25, and 100 µM) of each natural product were tested and used to determine the concentration, which resulted in 50% cell growth inhibition (IC_50_) (Table S8).

Staurosporine was used as a standard positive control, and its IC_50_ value was calculated in BT-474 and SKBR3 for comparison under the same experimental conditions with IC_50_ of 3.2 and 7.2 µM, respectively. Meanwhile, lapatinib was also included as a clinically approved HER2/EGFR tyrosine kinase inhibitor, and its IC_50_ value was determined (Table S8).

**Fig. 4:**
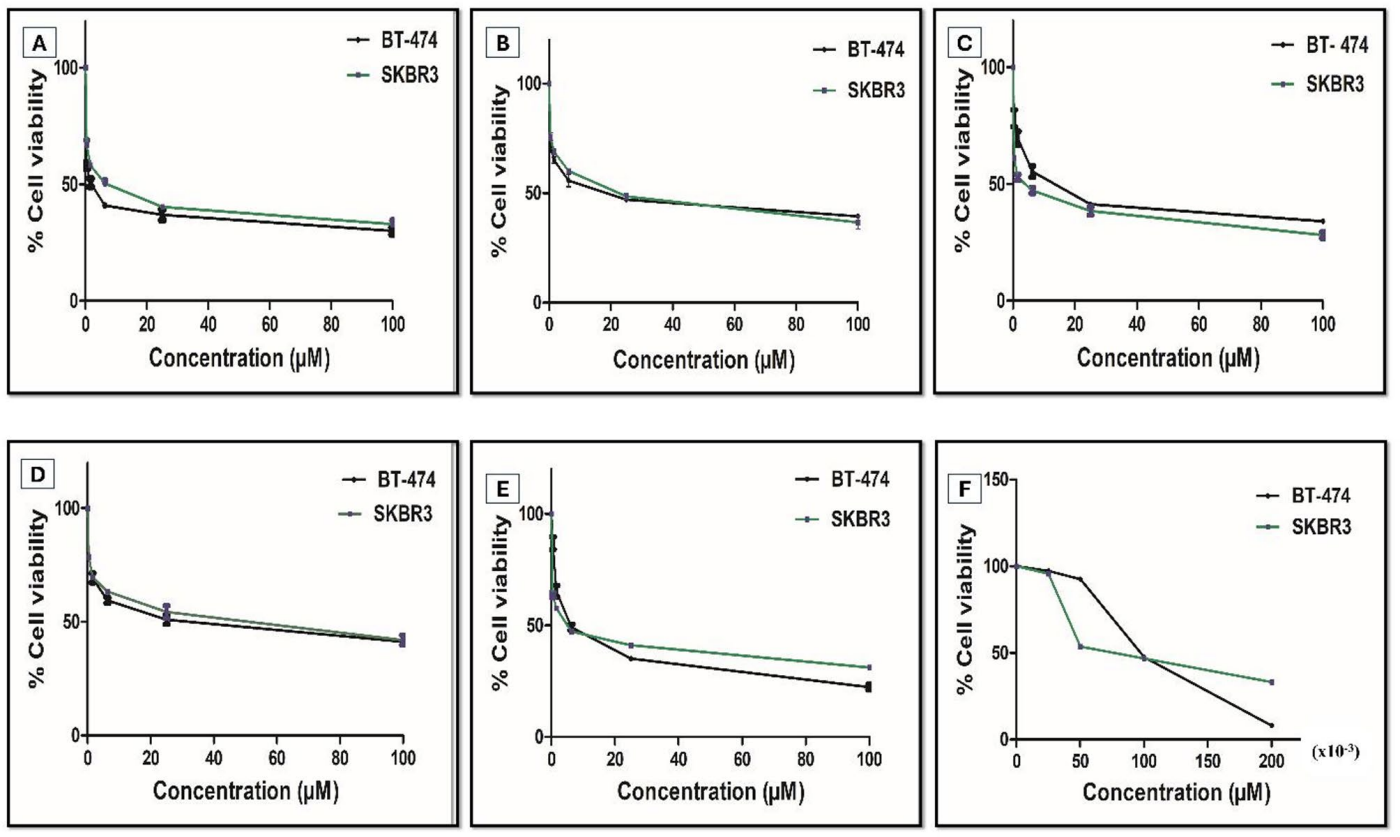
Effect of the potential hit’s treatment on HER2 over expressing BT-474 and SKBR3 breast cancer cells after 48 h treatment. MTT assay was used to determine viable cell count, Error bars represent the SD; (**A**) Liquiritin, (**B**) Oroxin B, (**C**) Mulberroside A, (**D**) Ligustroflavone, (**E**) Staurosporine and (**F**) Lapatinib.

In general, all tested compounds demonstrated comparable anti-proliferative activities to that of staurosporine against both breast cancer cell lines after 48 h of treatment (Fig. 4). The IC_50_ values of oroxin B, liquiritin, ligustroflavone, and mulberroside A in BT-474 cells were 6.9, 3.5, 6.0 and 5.9 µM, respectively while their IC_50_ values in SKBR3 cells were 11.9, 6.4, 12.7 and 11 µM, respectively. MTT assay results revealed that BT-474 cells were more sensitive towards treatment concentrations of all studied natural products, showing at least two-fold differences in their surviving fractions when compared to SKBR3 cells.

Liquiritin was the most potent in cell proliferation assay, being approximately two-fold more active than all tested natural products against the two HER2-positive human breast cancer cell lines investigated (Fig. 4). Although lapatinib exhibited greater absolute potency than liquiritin with IC_50_ values of 117.7 nM and 153 nM in BT-474 and SKBR3, respectively, our virtual screening-derived hit exhibited higher ligand efficiency (LE = 0.37 vs. 0.35 kcal/mol/heavy atom), a critical metric for early-stage drug discovery and a key predictor of optimization potential^63^.

Growing evidence in the literature highlights the anticancer potential of *Glycyrrhiza glabra* in general and its phytoconstituents in particular across different cancer types and specifically towards breast cancer. The ethanolic extract of *Glycyrrhiza glabra* root possessed significant antiproliferative effects against human mammary MCF-7 and hepatic HEPG2 cancer cells at 100 g/mL and 16.1 g/mL, respectively, with no effect being observed against human colon HCT-116 cancer at the same treatment doses^64^.

Hamta et al. showed the ability of the same extract to exhibit dose and time-dependent cytotoxic effects and morphological alterations in animal breast cancer 4T1 cells^65^. In a more recent report, the ethanolic extract of licorice roots inhibited the proliferation of MDA-MB-231 human breast cancer cells in a dose-responsive manner^66^. In this respect, several studies have established a robust correlation between the anti-breast cancer effects of *Glycyrrhiza glabra* and isoliquiritigenin as the metabolite with potential capacity to impede tumor angiogenesis, promote autophagy, induce apoptosis, and suppress cell proliferation^67,68^. Our molecular modeling, biochemical, and cellular data revealed that the potential anticancer effects of licorice are not limited to the presence of isoliquiritigenin and opens new directions for investigating liquiritin as a promising hit for the control of HER2-dependent breast cancer.

Results of the integrated in silico and in vitro workflow adopted in this study clearly revealed the ability of liquiritin to exhibit the most promising activity profile, considering the results of both primary and counter screens. It ranked second in cell-free assay while showing the highest activity in the cellular counter screen. The three remaining natural products demonstrated comparable efficacy in breast cancer cells, with oroxin B showing three-fold better activity in biochemical assay when compared to ligustroflavone and mulberroside A. Therefore, both liquiritin and oroxin B were considered for further evaluation as potential HER2 inhibitors, while ligustroflavone and mulberroside A have been excluded from additional testing.

#### Selective cytotoxic activity evaluation

It was essential to investigate the cytotoxic properties of liquiritin and oroxin B against the immortalized non-tumorigenic human MCF10A mammary epithelial cell line using an MTT assay. This assay aimed to assess the differential selectivity of chosen hits towards human breast cancer cell lines. Five different concentrations were used for each hit (0.4, 1.6, 6.3, 25, and 100 µM), and IC_50_ values were calculated to ensure proper selectivity evaluation (Table S9).

As compared to the vehicle-treated control group, investigated natural products did not significantly impact the viability of MCF10A cells at treatment doses equivalent to their corresponding IC_50_ values in human breast cancer cells (Fig. S6). Remarkably, oroxin B was shown to have an IC_50_ value of 53.0 ± 2.1 µM in MCF10A cells, which is 8 and 4.5 folds higher than its corresponding IC_50_ values in BT-474 and SKBR3 breast cancer cells, respectively. Meanwhile, liquiritin showed almost the same selectivity profile and exerted an IC_50_ value of 27.9 ± 1.1 µM in noncancerous cells, while it exhibited IC_50_ values of 3.5 and 6.4 µM in cell proliferation assay against BT-474 and SKBR3 cells, respectively (Table S9). Also, selectivity index (SI) for each compound against the two breast cancer cells varied, where based on IC_50_ in BT-474 cell line, SI of oroxin B and liquiritin were 7.7 and 8, respectively. While in the SKBR3 cell line, their SI were 4.5 and 4.4, respectively. Nevertheless, the high selectivity index values, > 3, indicated high selectivity towards cancerous cells and lower toxicity towards normal cells^69,70^ which allows a good safety margin from a therapeutic perspective. Together, these results implied that both liquiritin and oroxin B have a good selectivity margin towards HER2-dependent breast cancer cells, suggesting their potential as candidates to control this breast cancer phenotype.

#### Cell migration assays

**Fig. 5:**
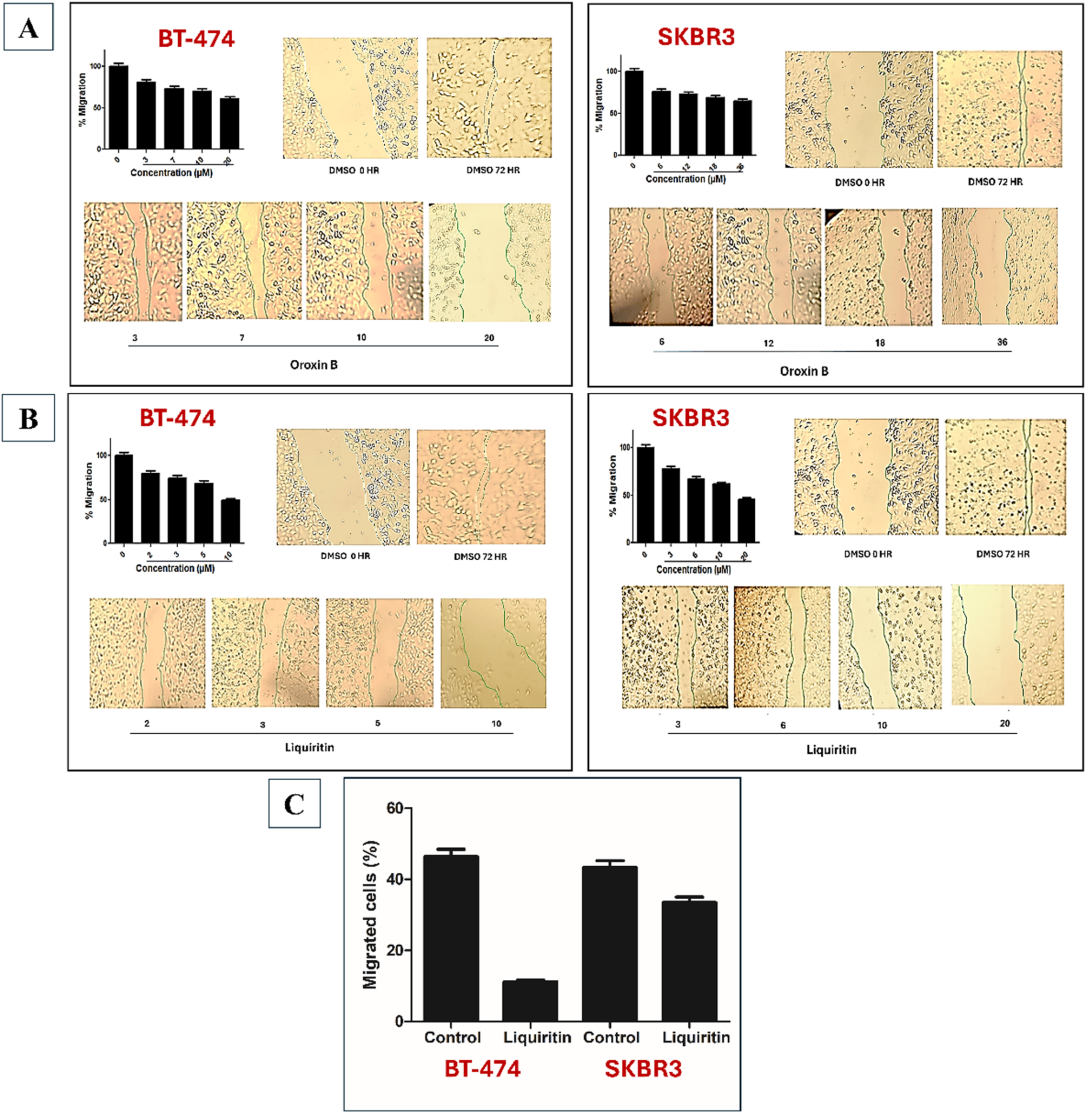
Effect of (**A**) Oroxin B and (**B**) Liquiritin on migration of left panel: BT-474 cells and right panel: SKBR3 cells in wound healing assay. Upper panel depicts quantitative analysis of the percentage of migration in various treatment groups in BT-474 cell line. Vertical bars show the percentage of wound closure at 72 h after wounding, calculated relative to the wound distance at zero time ± SD of *n* = 3 in each treatment group and Bottom panel shows photomicrographs of wound healing assay showing Oroxin B and Liquiritin treatment at four different concentrations as compared to DMSO as a vehicle control. (**C**) Vertical bar chart showing the % of migrated BT-474 and SKBR3 cells in transwell migration assay for control and liquiritin-treated groups.

Wound-healing assay is a simple in vitro approach to investigate directional cell migration in two dimensions. In the case of cancer cells, wound healing would simulate cancer cell migration, which takes place in vivo as a crucial step in metastasis. Treatment wells in this assay contained either liquiritin or oroxin B at four different doses, each in triplicate. In the case of BT-474 cells, treatment with 3, 7, 10, and 20 µM of oroxin B showed weak anti-migratory effects calculated as 18.9%, 26.5%, 29.5%, and 38.6% inhibition, respectively, as compared to the vehicle-treated control group (Fig. 5A). On the other hand, liquiritin significantly suppressed cell migration in a dose-dependent manner, with calculated values of 20%, 25.6%, 31.2% and 50.6% inhibition of cell migration at treatment concentrations of 2, 3, 5 and 10 µM, respectively (Fig. 5B). To ensure proper activity comparison, IC_50_ values were calculated, and as expected, liquiritin exerted an IC_50_ value of 10.4 µM. At the same time, it was impossible to calculate an IC_50_ for oroxin B at the chosen treatment doses due to its weak effects (IC_50_ > 20 µM). In case of SKBR3 cells, oroxin B showed roughly the same pattern of cell migration inhibition as in BT-474 cell line, with estimated percentage inhibition values of 23.5%, 26.5%, 31.1%, and 34.82% at 6, 12, 18, and 36 µM, respectively (Fig. 5A). Compared to DMSO-treated cells, liquiritin exerted a significant suppression of cell migration, with calculated values of 22.1%, 32.8%, 38.2% and 54.07% reduction after treatment with 3, 6, 10 and 20 µM, respectively (Fig. 5B). Apparently, liquiritin was more potent than oroxin B in SKBR3 cells; it showed an IC_50_ value of 16.9 µM in contrast to high micromolar anti-migratory activity of oroxin B (> 36 µM). Figure 5A and B displays bar charts and wound photographs from the cell migration assay, which demonstrate how the two compounds affected the migration of BT-474 and SKBR3 cells, compared to vehicle control at zero time and 72 h post-treatment.

The wound healing assay served as an initial screen to identify compounds disrupting collective cell migration in a monolayer. Liquiritin, which demonstrated robust inhibition of wound closure, was further evaluated in a transwell migration assay to quantify its ability to suppress directional chemotaxis, a critical feature of metastatic behavior. This two-step approach ensures both broad activity, as assessed by wound healing, and mechanistic specificity, as assessed by transwell migration, are validated, aligning with best practices for anti-migratory drug discovery.

Our results demonstrated that liquiritin treatment significantly reduced the migratory capacity of both BT-474 and SKBR3 cells compared to the negative control group (Fig. 5C). Treatment of BT-474 cells with 3.5 µM liquiritin for 24 h resulted in a marked reduction in migration, achieving approximately 76% inhibition relative to vehicle-treated cells. In comparison, SKBR3 cells exposed to 6.4 µM liquiritin for 24 h exhibited a more modest reduction in migration of approximately 23%. These results suggest that BT-474 cells are more sensitive to the anti-migratory effects of liquiritin than SKBR3 cells. Together, these findings support liquiritin’s potential as a candidate for further investigation in metastatic breast cancer, particularly in tumors with heightened HER2 activity. Future studies should prioritize elucidating the molecular mechanisms underlying this differential sensitivity, including liquiritin’s effects on cytoskeletal dynamics such as actin polymerization and key motility-related signaling pathways such as PI3K/AKT or FAK/Src.

### Biological characterization of liquiritin as a selective HER2 kinase inhibitor

#### Liquiritin modulates cell cycle dynamics and induces apoptosis in breast cancer cells

**Fig. 6:**
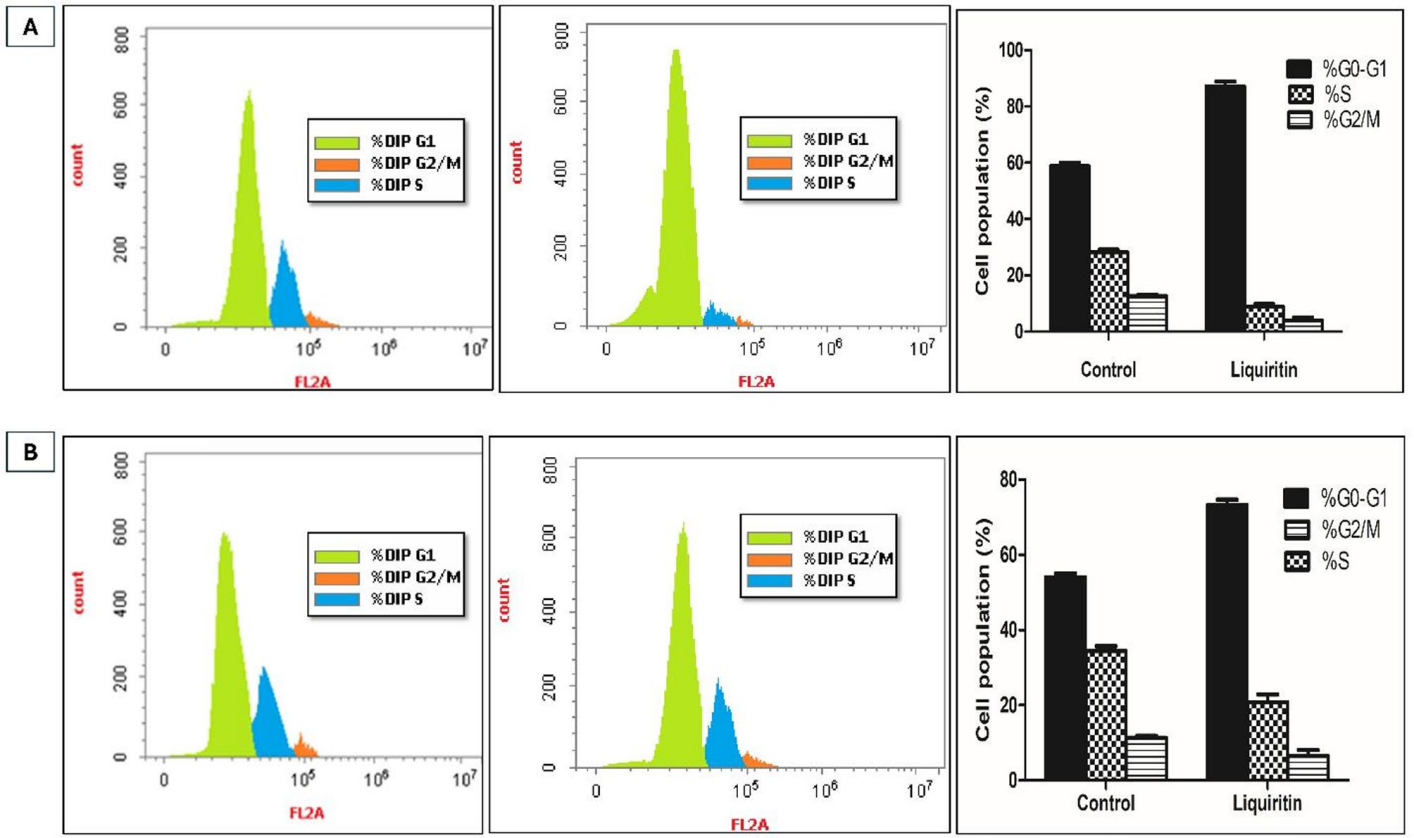
Flow cytometry analysis for (**A**) BT-474 and (**B**) SKBR3 cell cycle progression as represented by histograms of PI staining in control (left panel) and liquiritin-treated cells (middle panel). The right panel shows vertical bar charts representing the percentage of cells in each cell cycle phase of control and liquiritin-treated cells. Error bars represent the SD of three independent experiments.

To evaluate liquiritin’s effects on cell cycle progression, we analyzed DNA content via propidium iodide staining and flow cytometry in HER2-driven BT-474 and SKBR3 cells (Fig. 6). In BT-474 cells, treatment resulted in a robust increase in the proportion of cells in G1 phase of the cell cycle from 59% (vehicle-treated control) to nearly 88% with 3.5 µM liquiritin (Fig. 6A). Similarly, SKBR3 cells treated with 6.4 µM liquiritin exhibited a significant rise in G1 phase occupancy from 54 to 73% (Fig. 6B). These findings demonstrated that low micromolar doses of liquiritin effectively halt cell cycle progression at the G1 checkpoint, contributing to its growth-inhibitory activity in HER2-positive mammary cancer cells.

**Fig. 7:**
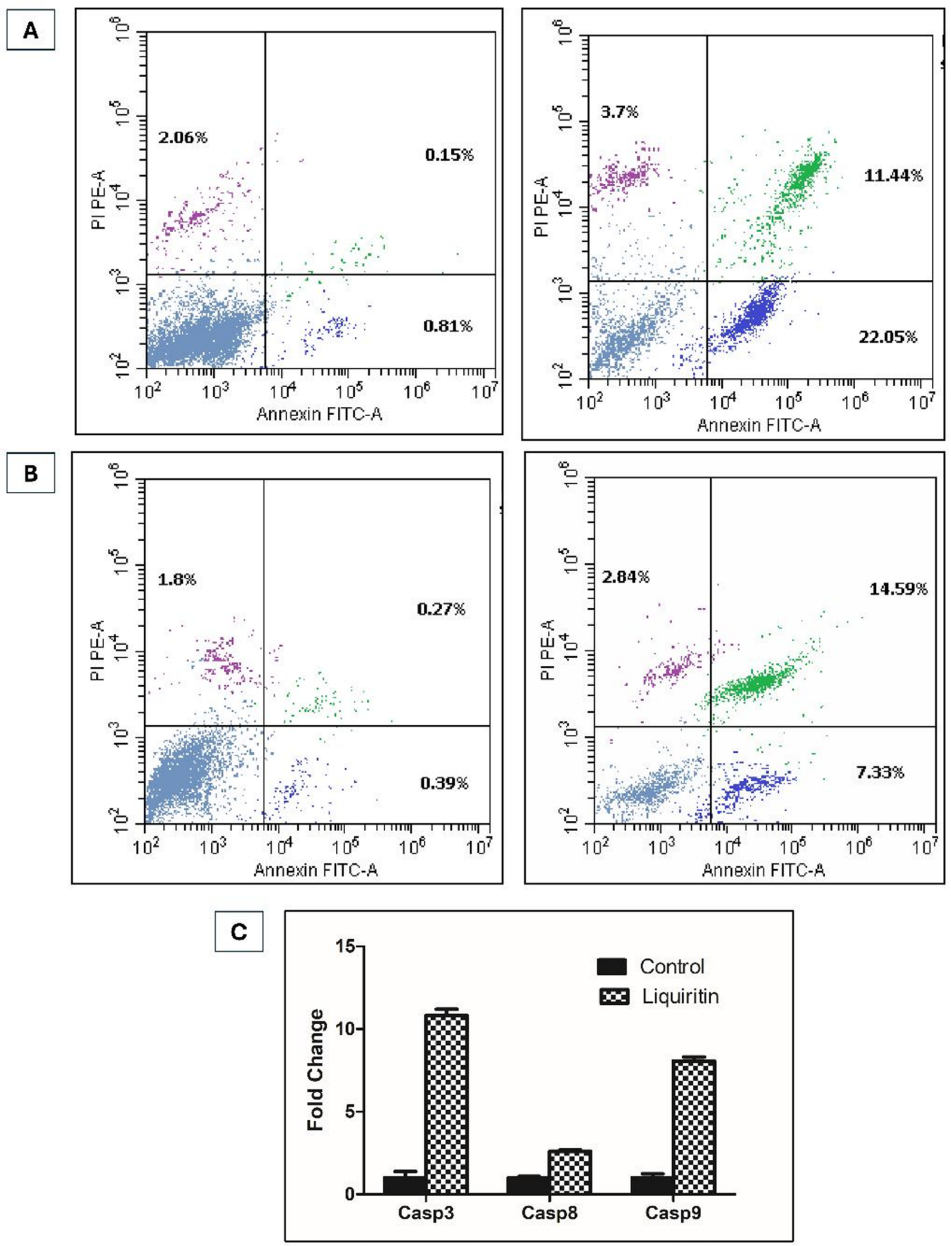
Double variable flow cytometry dot plots for (**A**) BT-474 and (**B**) SKBR3 cells analyzing pro-apoptotic effects of control (left panel) and liquiritin-treated cells (right panel) using Annexin V-FITC and propidium iodide (PI) dual staining. LL quadrant (FITC^−^/PI^−^) represents living cells; LR quadrant (FITC^+^/PI^−^) shows early apoptotic cells; UL quadrant (FITC^−^/PI^+^) depicts necrotic cells; and UR quadrant (FITC^+^/PI^+^) denotes late apoptotic cells. (**C**) Effect of liquiritin on the expression of caspases 3, 8, and 9 in BT-474 cells. Vertical bars represent fold change of expression ± SD as compared with untreated cells.

Given the established link between cell cycle arrest and apoptotic induction, the pro-apoptotic effects of liquiritin in HER2-positive mammary cancer cells were investigated. BT-474 and SKBR3 cells were treated with liquiritin at 3.5 µM and 6.4 µM, respectively, for 48 h. Cell death was assessed post-treatment via Annexin V (apoptotic marker) and propidium iodide (PI, necrotic marker) dual staining using flow cytometry (Fig. 7A and B).

In BT-474 cells, 3.5 µM liquiritin preferentially triggered early apoptosis, with 22.05% of cells staining Annexin V^+^/PI^−^, a 2.7-fold increase over baseline, compared to 11.44% in late apoptosis (Annexin V^+^/PI^+^) and minimal necrosis (3.7%, Fig. 7A). In contrast, SKBR3 cells treated with 6.4 µM liquiritin exhibited moderate late apoptosis (14.59% Annexin V^+^/PI^+^) alongside 7.33% early apoptotic and 2.84% necrotic populations (Fig. 7B). These results align with liquiritin’s IC_50_ values from proliferation assays, underscoring its dual role in halting cell cycle progression and initiating programmed death in a cell line-specific manner.

The differential apoptotic responses, early apoptosis in BT-474 versus late apoptosis in SKBR3, may reflect variations in HER2 expression levels or downstream signaling. The preferential induction of early apoptosis in BT-474 cells, which exhibit higher HER2 amplification, suggests that liquiritin likely targets HER2-driven survival pathways, such as PI3K/AKT signaling, thereby activating intrinsic apoptotic mechanisms^71,72^. Heightened HER2 amplification in BT-474 cells could sensitize them to liquiritin-mediated mitochondrial depolarization or caspase-9 activation^71,73^. In contrast, SKBR3 cells, which exhibit moderate HER2 dependency, may undergo slower apoptotic turnover, delaying caspase activation and favoring late apoptotic or necrotic outcomes upon liquiritin treatment^71,73^. This mechanistic distinction is consistent with established models linking HER2 pathway activity to apoptotic sensitivity and cell death kinetics in breast cancer cells.

#### Liquiritin prompts caspase-mediated apoptosis

Cells commence programmed cell death through two main routes, namely the intrinsic and extrinsic apoptosis pathways. Caspases are a group of enzymes that play a vital role in apoptosis. The intrinsic route can be triggered through caspase-9 and the extrinsic route is mediated by caspase-8. Both routes initiate apoptosis via cleavage of caspase-3, a central downstream executioner protein, that can interact with both caspases 8 and 9 ^74,75^. Caspases 3 and 9 expression levels were significantly higher in BT-474 cells treated with liquiritin showing approximately 10.8-fold and 8.1-fold higher expression compared to untreated cells (Fig. 7C). On the other hand, expression of caspase-8 showed only a 2.6-fold increase. These results confirmed caspase-mediated apoptosis triggered by liquiritin involving mainly the intrinsic pathway through overexpression of caspase-9 with less contribution of caspase-8 controlled extrinsic pathway.

#### Effect of liquiritin on HER2 phosphorylation

**Fig. 8:**
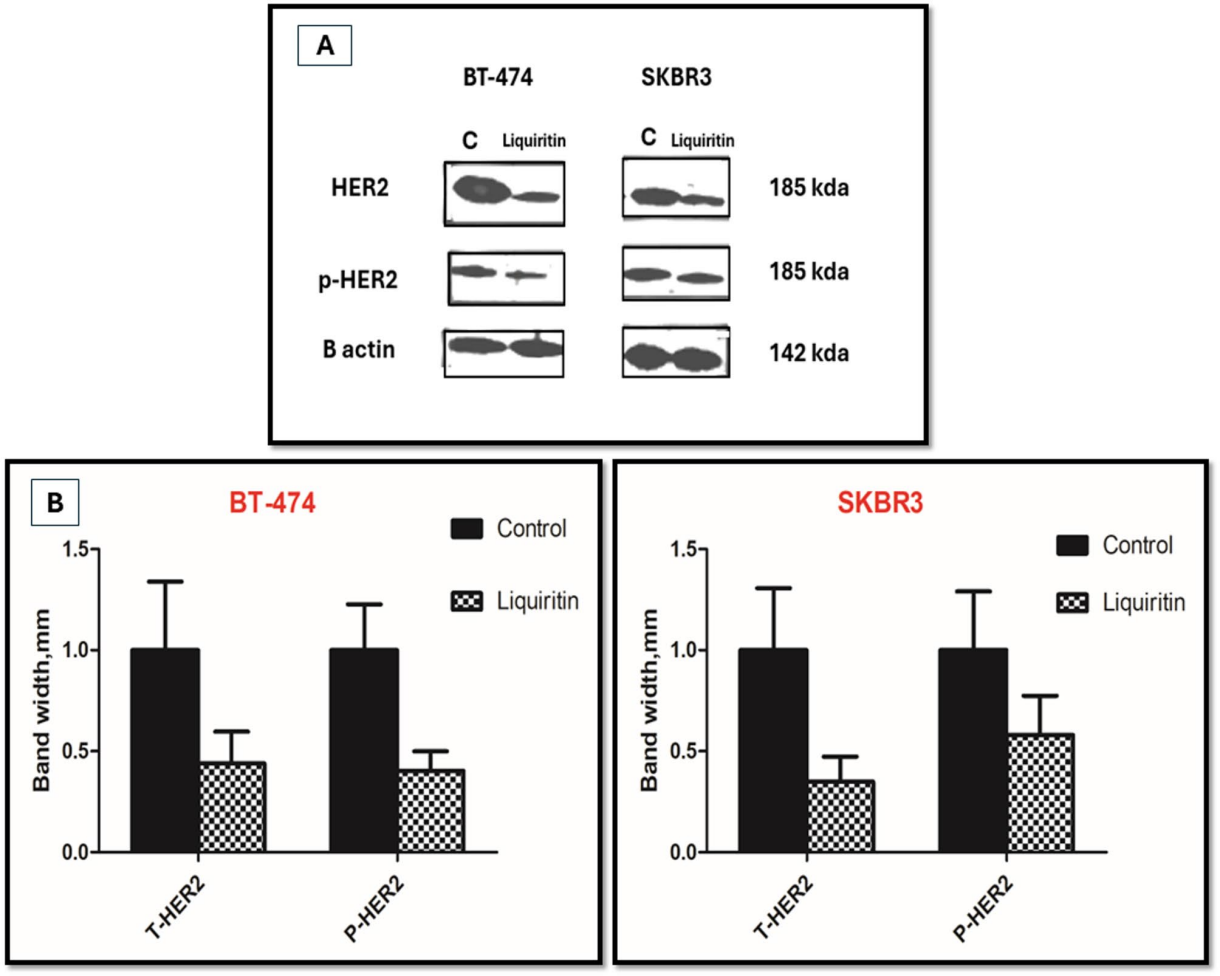
(**A**) Western blots images and (**B**) bar graphs in treatment on left panel for BT-474 and Right panel for SKBR3 cell lines representing summary densitometric data showing the effect of liquiritin on catalysis of HER2 protein and its total levels after treatment for 72 h, each blot was conducted in triplicate and the integrated optical density of each band was normalized with corresponding β-actin. Vertical bars in the graph denote the normalized integrated optical density of bands visualized in each lane ± SD as compared with vehicle-treated controls. Original blots are presented in (Supplementary Fig. S7).

As shown earlier, liquiritin exhibited the best overall profile as a HER2 inhibitor, which is evidenced by its nanomolar efficacy in cell-free assay and its potent anti-proliferative effects towards HER2-driven breast cancer cell lines. In an attempt to confirm the ability of liquiritin to inhibit HER2 catalysis on the molecular level, Western blot analysis was performed. In this study, both BT-474 and SKBR3 breast cancer cell lines were used to evaluate the effects of liquiritin on the total HER2 protein levels as well as its phosphorylation (catalysis). BT-474 and SKBR3 cells were treated with 5 and 10 µM doses of liquiritin, respectively. Meanwhile, cells in the control group were treated with DMSO as a vehicle. Following that, the levels of HER2 expression and phosphorylation were assessed in cell lysates. As shown in Fig. 8, liquiritin significantly inhibited HER2 phosphorylation in both BT-474 and SKBR3 cell lines compared to vehicle-treated control groups and β-actin, which was used as a loading control. Interestingly, liquiritin showed comparable suppressive effects on the total expression levels of HER2 protein following 72 h treatment at the tested concentrations.

Western blotting clearly validated the results of our virtual screening campaign and revealed the ability of liquiritin to inhibit not only the activation of HER2 protein through binding to its kinase catalytic site but also its total expression levels. These findings implied the potential of liquiritin to act upon HER2 kinase through multiple complementary mechanisms rather than acting only as an ATP competitive kinase inhibitor. Interestingly, recent reports corroborated our findings, showing a similar promiscuous pattern for the structurally related aglycone, isoliquiritigenin, in breast cancer cells^76^. Isoliquiritigenin directly inhibited the kinase activity of vascular endothelial growth factor receptor-2 (VEGFR-2) and enhanced the proteasomal degradation of HIF-1, hence limiting the expression of its ligand, vascular endothelial growth factor (VEGF), in breast cancer cells^76^. Another study unveiled the mode of action of isoliquiritigenin in downregulating the co-expression of HER2 and HER3 proteins in breast cancer at least in part through the inhibition of PI3K-AKT- mTOR signaling pathway. Such downregulation ultimately resulted in a significant reduction of breast cancer cells proliferation^77^. Additionally, liquiritin is a flavonoid similar to quercetin and apigenin. It has been reported that quercetin induced HER2 downregulation through the proteasome degradation pathway^78^ and likewise, apigenin reduced HER2 levels by inducing its degradation in the proteasome^79^. Hence, it is similarly suggested that HER2 downregulation by liquiritin does not occur at the transcriptional level but post-translation due to degradation. To conclusively rule out transcriptional effects, future work will include qPCR analysis of HER2 mRNA in liquiritin-treated cells. Together, these results open future directions for further investigation of the multidimensional molecular mechanisms underlying the effects of liquiritin in HER2-dependent breast cancer.

#### Selectivity assessment of liquiritin

The exceptional ability of liquiritin to block ATP binding and HER2 auto-phosphorylation encouraged us to explore its selectivity profile against a panel of tyrosine kinases that are structurally and oncogenically related to HER2. Liquiritin was screened at 0.1 µM dose against a subset of 15 tyrosine kinases using the Select Screen Kinase Profiling Service. As shown in Table S10, the assay provided results as % inhibition of each kinase with very high Z´-factor values (> 0.8). Interestingly, 0.1 µM of liquiritin induced potent inhibition (> 80%) against the ErB family of receptor tyrosine kinases, including HER2, EGFR (ERBB1), and ERBB4 (HER4), with EGFR exhibiting the greatest relative inhibition. Meanwhile, liquiritin scarcely inhibited the activity of the other tested tyrosine kinases, in contrast to its considerable effectiveness against HER2 kinase and the other ERBB family members. In this respect, Liquiritin was found to be 11 to 12 folds selective for the ERBB family versus other tested kinases. According to this preliminary kinase profiling study, liquiritin exhibited exquisite selectivity towards the ERBB family at a pharmacologically relevant concentration.

Our initial profiling raised the potential that liquiritin could be a promising EGFR/HER2/ERBB4 triple kinase inhibitory hit or Pan HER inhibitor. Such discovery might provide an additional advantage for liquiritin as a HER2 kinase inhibitor. Its profound selectivity towards a small number of closely related disease-critical kinases would offer a broader scope of therapeutic benefits whilst lacking potential off-target toxicity of poorly selective kinase inhibitors. For instance, liquiritin showed high potency against EGFR besides HER2 kinases, which might suggest its potential to act upon the EGFR-overexpressing triple-negative breast cancer in addition to HER2-driven phenotypes.

**Fig. 9:**
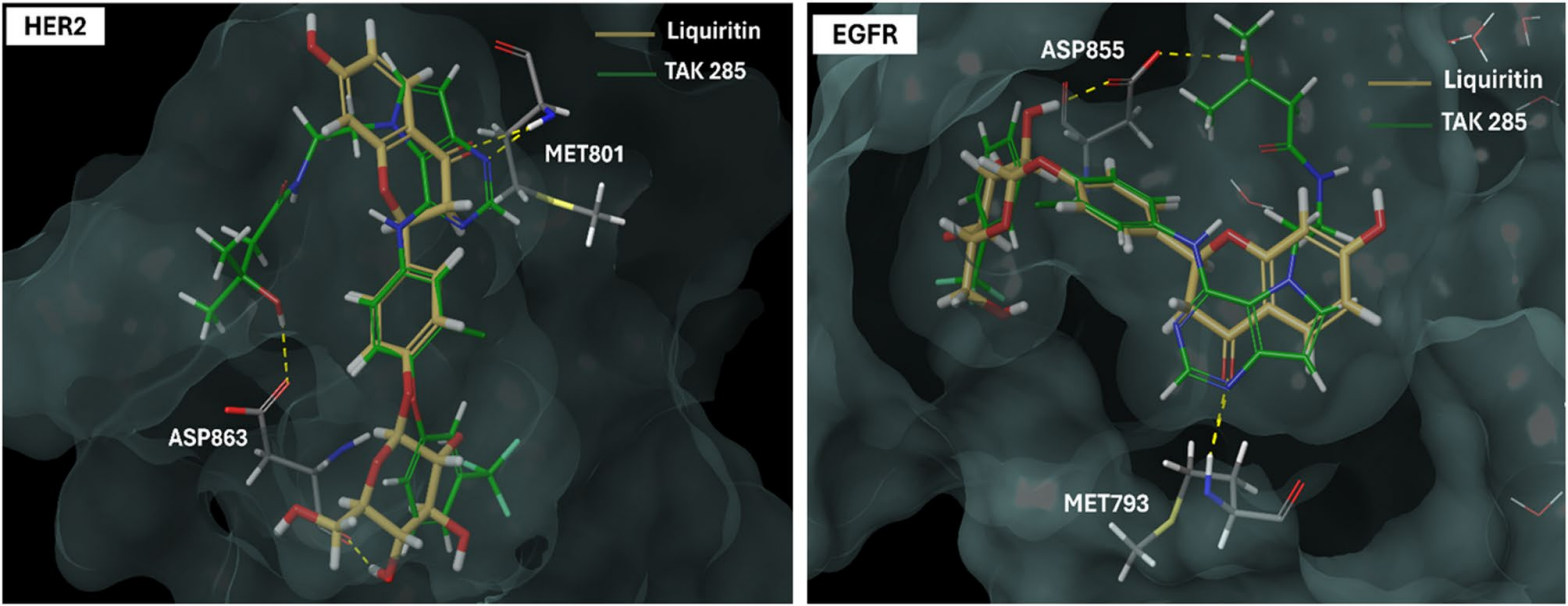
In silico binding mode of liquiritin at the ATP binding site of HER2 and EGFR kinase domains. Left panel, structure overlay for liquiritin (yellow) with TAK 285 conformations (green) obtained from EGFR crystal structure 3POZ. Right panel, structure overlay for liquiritin (yellow) with TAK 285 conformations (green) obtained from HER2 crystal structure 3RCD.

Further docking simulation experiments have been conducted for liquiritin against EGFR kinase using Glide extra-precision (XP) (Schrödinger 2014). Docking studies attempted to analyze liquiritin binding mode within EGFR kinase in comparison with its corresponding HER2 pose. In addition, liquiritin was subjected to overlay studies with TAK 285 as a standard EGFR/HER2 dual inhibitor in an attempt to reveal the structural basis for liquiritin’s HER family selectivity. Figure 9 illustrated the characteristic interactions of liquiritin towards the HER family and showed its perfect overlay with TAK 285 within the binding site of each kinase. Binding mode analyses revealed the amino acid residue in charge of liquiritin’s HER family selectivity. The aspartate residues 863 and 855, which are positioned in the DFG motif of HER2 and EGFR, respectively, were suggested to be responsible for selectivity.

## Conclusion

Nature is a source of abundant small molecules that can be lead compounds as receptor inhibitors in breast cancer-targeted therapy. One of the promising prospects for HER2-positive breast cancer targeted therapy is the identification of novel lead compounds from natural sources that target the HER2 tyrosine kinase domain. Through a structure-based virtual screening campaign, this study analyzed an extensive virtual library that contains roughly 638.960 natural products for possible binding to the HER2 tyrosine kinase domain. Five of these natural products: oroxin B, liquiritin, ligustroflavone, mulberroside A, and delphin were selected for biological validation. The first four compounds demonstrated low nanomolar cell-free activities comparable to lapatinib in the primary screen, and promising low micromolar anti-proliferative actions against HER2 overexpressing breast cancer cell lines in the counter screen. Among the four compounds, liquiritin revealed a promising anti-migratory activity in two different cellular motility models. Other tested hits had weak anti-migratory activities and mainly affected cancer cell growth rather than development and metastasis. Using a variety of biochemical and cellular assays, along with molecular dynamics simulations, liquiritin stood out as a putative HER2 inhibitor, significantly suppressing both HER2 phosphorylation and expression in breast cancer cells. In addition, liquiritin induced G1 cell cycle arrest and proapoptotic effects in HER2-driven mammary cancer cells. Apoptosis occurred primarily through caspase-9–mediated intrinsic signaling, with limited extrinsic pathway involvement via caspase-8.

Importantly, liquiritin demonstrated notable selectivity for HER family proteins when tested against various kinases, highlighting its potential as a promising pan-HER inhibitor candidate for future development. This study also provided preliminary SAR insights for effective HER2 inhibition that would guide future optimization campaigns of liquiritin. Our results also highlighted the importance of virtual screening as an effective tool for deorphanization of natural products. Additionally, this study breaks new ground by demonstrating that liquiritin, a component of licorice, shows promise in combating HER2-positive breast cancer, expanding the understanding of licorice’s anticancer properties beyond isoliquiritigenin.

Compared to approved therapies available in the market, liquiritin has several advantages, as it suppresses HER2 at nanomolar levels, selectively targets the HER family (EGFR, HER2, and HER4), and has a negligible effect on non-cancerous cells. Due to its natural nature, liquiritin may provide a wider safety margin and broader HER family inhibition with possibly fewer off-target effects than lapatinib or neratinib. Despite lower absolute potency, liquiritin exhibited competitive ligand efficiency compared to lapatinib. Meanwhile, virtual screening aims to identify tractable hits/leads with novel chemotypes, not final drugs. Early-stage hits should prioritize diversity and optimizability over direct potency comparisons to approved drugs. Lapatinib itself originated from an initial micromolar hit, which was optimized over several years to achieve nanomolar potency^80^. Liquiritin’s promising efficacy also suggests its potential as an adjuvant to existing therapies, particularly given its favorable in silico ADME/Tox profile. Its natural origin, lower cost, and HER inhibitory potential make it an attractive candidate for further drug development. To support the hit-to-lead advancement of our findings, additional in vivo evaluation of this natural product is still required, and it is currently being addressed in follow-up studies after scale-up production of liquiritin. These studies particularly using human HER2 transgenic mouse models are crucial to evaluating its efficacy in suppressing HER2-driven tumorigenesis and overcoming resistance to existing HER2 inhibitors, in addition to validating its pharmacokinetic and toxicity profiles. The promising activity of liquiritin may guide the development of novel and potent HER2 inhibitors.

## Electronic supplementary material

Below is the link to the electronic supplementary material.

Supplementary Material 1

Supplementary Material 2

## Data Availability

Data generated during this study is provided within the manuscript and supplementary information files. Additional data will be made available upon reasonable request to the corresponding author.
