# Supplementary material for: Discovery of natural scaffolds as HER2 inhibitors for breast cancer: virtual screening, molecular dynamics, and biological characterization with selectivity profiling
Source: Sci Rep. 2025 Jul 17;15:25883. doi: 10.1038/s41598-025-11177-6 (PMC12267471; doi:10.1038/s41598-025-11177-6)
Supplement: Supplementary file 2 — Supplementary Material 2 [file 41598_2025_11177_MOESM2_ESM.pdf]

Supplementary information

Discovery of natural scaffolds as HER2 inhibitors for breast cancer: Virtual screening, molecular dynamics, and biological characterization with selectivity profiling

Asmaa Hossam[a], Ingy I. Abdallah[a,*], Nadia A. El-Sebakhy[a], Radwan Alnajjar[b], Mohamed M. Mohyeldin[a]

[a] Department of Pharmacognosy, Faculty of Pharmacy, Alexandria University, Egypt.
[b] Department of Chemistry, Faculty of Science, University of Benghazi, Benghazi, Libya.

[*] Corresponding author: Dr. Ingy I. Abdallah, Alkhartoom Square, Department of Pharmacognosy, Faculty of Pharmacy, Alexandria University, Alexandria 21521, Egypt.
E-mail: ingy.ibrahim@alexu.edu.eg
Fax: +2034871668-4873273

Table S1. Structures and names of the training set and their molecular targets.

#	Drug	Structure	Chemical class	Inhibition target
**1**	**Lapatinib**	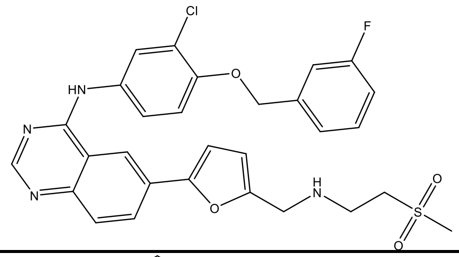	**4-anilinoquinazoline**	Dual TKI for HER2 and EGFR.[1]
**2**	**Neratinib (HKI-272)**	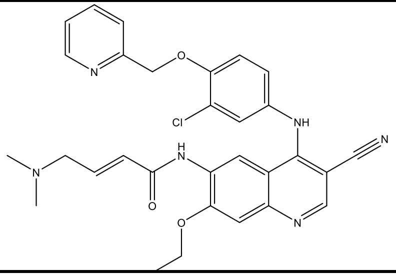	**Quinoline**	Irreversible Pan-HER (EGFR or HER1), HER2, and HER4.[1]
**3**	**Afatinib (BIBW 2992)**	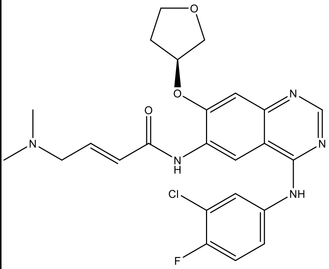	**Quinazolines**	**Irreversible EGFR, HER-2.1**
**4**	**BMS-599626**	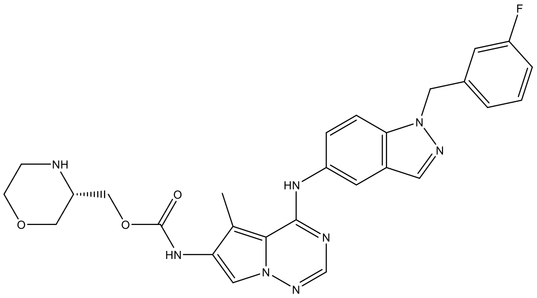	**Carbamates**	Reversible EGFR, HER2, and HER4 inhibitor.[1,2]
**5**	**CP-724714**	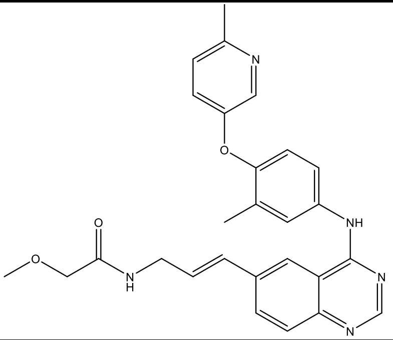	**Quinazolinamines**	HER2-specific TKI.[1]

**6**	**AEE788**	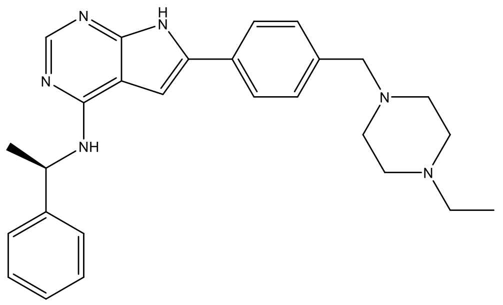	**Phenylpyrroles**	Dual specific reversible EGFR and HER2 kinase inhibitor.[1,2]
**7**	**Gefitinib (ZD1839)**	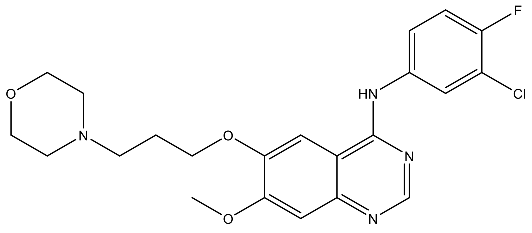	**Quinazolinamines**	Inhibits breast cancer cells overexpressing HER2 *In-Vitro* and *in-Vivo* studies.[3]
**8**	**Sorafenib**	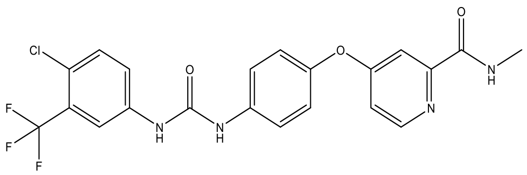	**Diaryl ethers**	Multi-kinase inhibitor.[4]
**9**	**Epertinib (S-222611)**	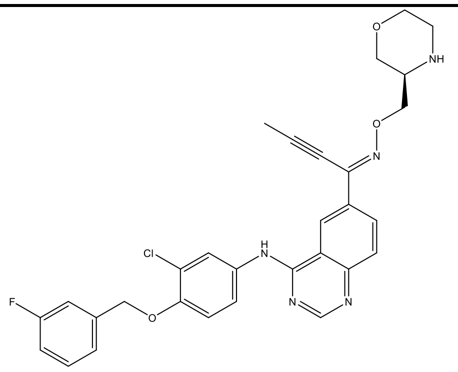	**Quinazolines**	Reversible inhibitor of EGFR, HER2 and HER4 receptor tyrosine kinases.[5]
**10**	**Tucatinib**	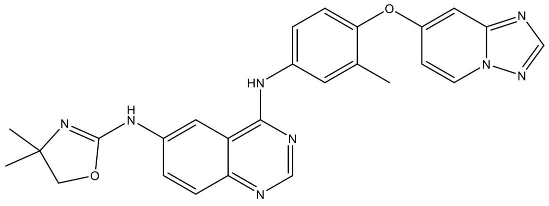	**Pyridines**	EGFR and HER2.[6]

**11**	**Poziotinib**	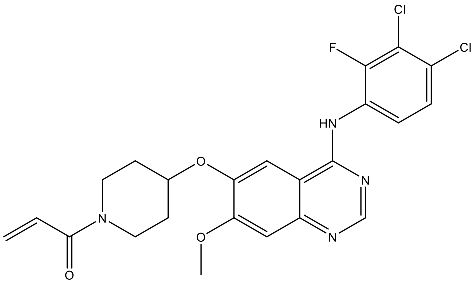	**Quinazolines**	Irreversible inhibitor of EGFR, HER2 and HER4.[7]
**12**	**Canertinib (CI-1033)**	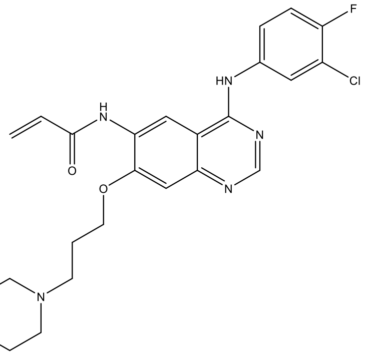	**Morpholines**	Irreversible EGFR, HER2, and HER4 tyrosine kinase inhibitor.[2,8]
**13**	**Pyrotinib**	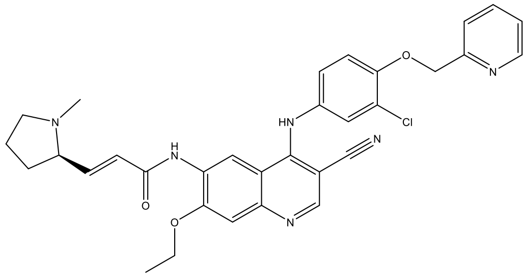	**Acrylamides**	Irreversible EGFR, HER2, and HER4 tyrosine kinase inhibitor.[6,9]
**14**	**Dacomitinib**	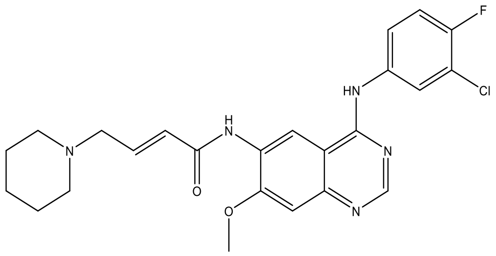	**Quinazolinones**	Irreversible inhibitor of the ERBB family of kinases: EGFR, ERBB2, and ERBB4 with IC_{50}s 6 nM, 45.7 nM & 73.7 nM, respectively.[10]

**15**	**Sapitinib (AZD-8931)**	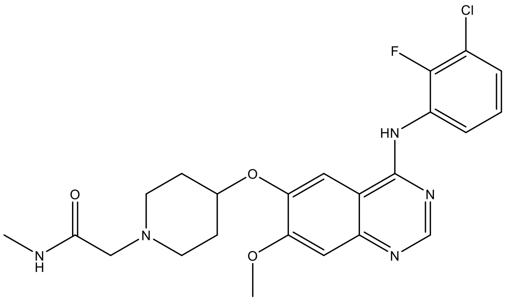	**Quinazolines**	Reversible inhibitor of EGFR, HER2, and HER3.[2]
**16**	**Allitinib (AST-1306)**	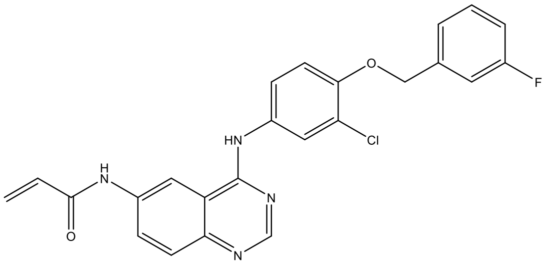	**Acrylamides**	Irreversible EGFR and ErbB2 inhibitor.[2]
**17**	**CUDC-101**	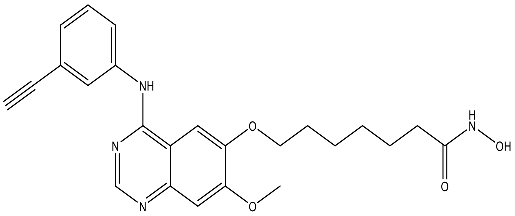	**Hydroxamic Acids**	An irreversible EGFR and HER2 inhibitor.[2]
**18**	**Pelitinib (EKB-569)**	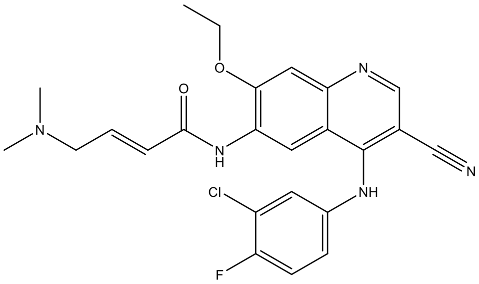	**Aniline Compounds**	Irreversible dual EGFR/HER2 inhibitor.[2]

Table S2. Primers used for real-time PCR

Gene	Forward primer	Reverse primer
Caspase 3	5’-GGAAGCGAATCAATGGACTCTGG-3’	5'-GCATCGACATCTGTACCAGACC-3'
Caspase 8	5’-AGAAGAGGGTCATCCTGGGAGA-3’	5'-TCAGGACTTCCTTCAAGGCTGC-3'
Caspase 9	5’-GTTTGAGGACCTTCGACCAGCT-3’	5’-CAACGTACCAGGAGCCACTCTT-3’
GAPDH	5’-GTCTCCTCTGACTTCAACAGCG -3’	5’-ACCACCCTGTTGCTGTAGCCAA-3’

Table S3. Validation parameters of molecular docking for 3RCD crystal structure.

Validation parameters	3RCD
RMSD*	**0.3**
AUC-ROC	**0.99**
EF (2%)	**52**
EF (5%)	**20**
EF (10%)	**10**
RIE	**16.85**
BEDROC ($\alpha = 8$)	**1**
BEDROC ($\alpha = 20$)	**1**
BEDROC ($\alpha = 160$)	**1**
Ranked actives**	**18**
Approximate sensitivity	**0.99**
Specificity	**0.98**

*RMSD value was calculated for 3RCD HER2 crystal structure with co-crystallized ligand (TAK-285)
**Ranked actives are the number of actives recovered from the constructed validation set.

Table S4. The docking scores of the validation set.

Ranking	Compound	Docking score
**1**	**Lapatinib**	-12
**2**	**Epertinib**	-11.4
**3**	**Allitinib**	-10.9
**4**	**CP-724714**	-10.7
**5**	**CUDC-101**	-10.7
**6**	**BMS-599626**	-9.5
**7**	**Tucatinib**	-9.2
**8**	**Neratinib**	-8.7
**9**	**Poziotinib**	-8.5
**10**	**Sapitinib**	-8.2
**11**	**Gefitinib**	-7.5
**12**	**Canertinib**	-7.4
**13**	**Pyrotinib**	-6.7
**14**	**AEE788**	-6.3
**15**	**Pelitinib**	-6
**16**	**Sorafenib**	-5.8
**17**	**Afatinib**	-5.7
**18**	**Dacomitinib**	-5.2

Table S5. ADME and drug-likeness attributes of the four active hits by QikProp.

Name	# stars	mol_MW1[a]	QPPCaco[b]	QPlogKhsa[c]	Percent Human Oral Absorption[d]	RO5[e]	RO3[f]	QPlogBB[h]	QPlogHERG[i]	#metab[j]
Oroxin B	9	594.525	1	-1.415	0	3	2	-5.26	-7	9
Mulberroside A	11	568.53	0.5	-1.636	0	3	2	-6.14	-7	10
Liquiritin	0	418.399	23	-0.723	50	0	2	-2.82	-5	7
Ligustroflavone	13	724.668	0.6	-1.741	0	3	2	-5.87	-7	10

(a) Molecular weight (mol_MW) (130−725), (b) Apparent Caco-2 cell permeability (QPPCaco) (nm/s; <25 poor, >500 great), (c) QPlogKhsa (a Schrödinger's QSAR-based descriptor for human serum albumin binding) (-1.5 to 1.5) and (d) percent human oral absorption (≥80% is high, ≤25% is poor). (e) RO5: number of violations of Lipiniski's rule of five[8]. The accepted range: maximum is 4. (f) RO3: Number of violations of Jorgensen's rule of three[9]. Accepted range: maximum is 3. (h) BBB partition coefficient (QPlogBB) (-3 to 1.2). (i) Predicted IC_{50} value for blockage of HERG K^+ channels (QPlogHERG) (not below -5). (j) Number of likely metabolic reactions (#metab) (1-8).

Table S6. The physicochemical properties of liquiritin estimated using the SwissADME database.

Name	MW[a] (g/mol)	Lipophilicity	Water solubility						Bioavailability Score
		Consensus Log P	Log S (ESOL)	solubility Class	Log S (Ali)	solubility Class	Log Sw (SilicosIT)	solubility Class	
Liquiritin	418.39	0.25	-2.71	Soluble	-3.02	Soluble	-2.29	Soluble	0.55

(a) MW: molecular weight

Table S7. The Prime MM-GBSA energies of Oroxin B and Liquiritin binding in the active site of HER2.

Complex	ΔG Binding	Coulomb	Covalent	H-bond	Lipo	Bind packing	Solv_GB	vdW	SD
**Oroxin-HER2**	-51.41	-22.23	1.31	-1.58	-12.61	-0.31	24.62	-40.61	3.64
**Liquiritin-HER2**	-63.70	-30.83	2.21	-3.71	-14.14	-0.20	28.20	-45.23	6.21

Coulomb: Coulomb energy; Covalent: Covalent binding energy; H-bond: Hydrogen-bonding energy; Lipo: Lipophilic energy; Solv_GB: Generalized born electrostatic solvation energy; vdW: van der Waals energy; SD: Standard deviation.

Table S8. IC_{50} values of potential hits against SKBR3 and BT-474 cell lines overexpressing HER2 protein.

Compounds	Cell growth inhibition IC_{50} (μM)	
	BT-474	SKBR3
Liquiritin	3.5 ± 0.1	6.4 ± 0.2
Mulberroside A	5.9 ± 0.5	11.0 ± 0.1
Oroxin B	6.9 ± 0.7	11.9± 0.8
Ligustroflavone	6.0 ±1.1	12.7± 1.5
Staurosporine	3.2 ± 0.3	7.2 ± 0.2
Lapatinib	0.117 ± 2.3	0.153 ± 3.3

Table S9. IC_{50} values of Oroxin B and Liquiritin in BT-474, SKBR3 and MCF10A cells.

Compound	IC_{50} (μM)		
	BT-474	SKBR3	MCF10A
Oroxin B	6.9 ± 0.7	11.9 ± 0.8	53.0 ±2.1
Liquiritin	3.5 ± 0.1	6.4 ± 0.2	27.9 ±1.1
Staurosporine	3.2 ± 0.3	7.2 ± 0.2	26.5 ±1.0

Table S10. Initial TK-selectivity profile of Liquiritin.

Kinase	Mean inhibition (%)	Z'-Score
**ALK**	7	0.89
**EGFR (ErbB1)**	**99**	0.88
**ERBB2 (HER2)**	**92**	0.83
**ERBB4 (HER4)**	**88**	0.92
**FGFR1**	7	0.89
**FLT1 (VEGFR1)**	2	0.90
**IGF1R**	4	0.86
**KDR (VEGFR2)**	4	0.92
**KIT**	0	0.93
**MET (cMet)**	5	0.81
**MST1R (RON)**	7	0.92
**PDGFRB (PDGFR beta)**	4	0.90
**RET**	2	0.92
**ROS1**	8	0.86
**TYRO3 (RSE)**	5	0.92

ALK, anaplastic lymphoma kinase; ErbB, erythroblastic leukemia viral oncogene homolog; FGFR1, fibroblast growth factor receptor 1; FLT1, fms-like tyrosine kinase; KDR, kinase insert domain receptor; RON, recepteur d'origine Nantais; MST1R, macrophage stimulating 1 receptor; PDGFR, platelet-derived growth factor receptor; RET, Multiple Endocrine Neoplasia and Medullary Thyroid Carcinoma 1; ROS1, v-ros avian UR2 sarcoma virus oncogene homolog 1; TYRO3, tyrosine protein kinase; RSE, receptor sectatoris.

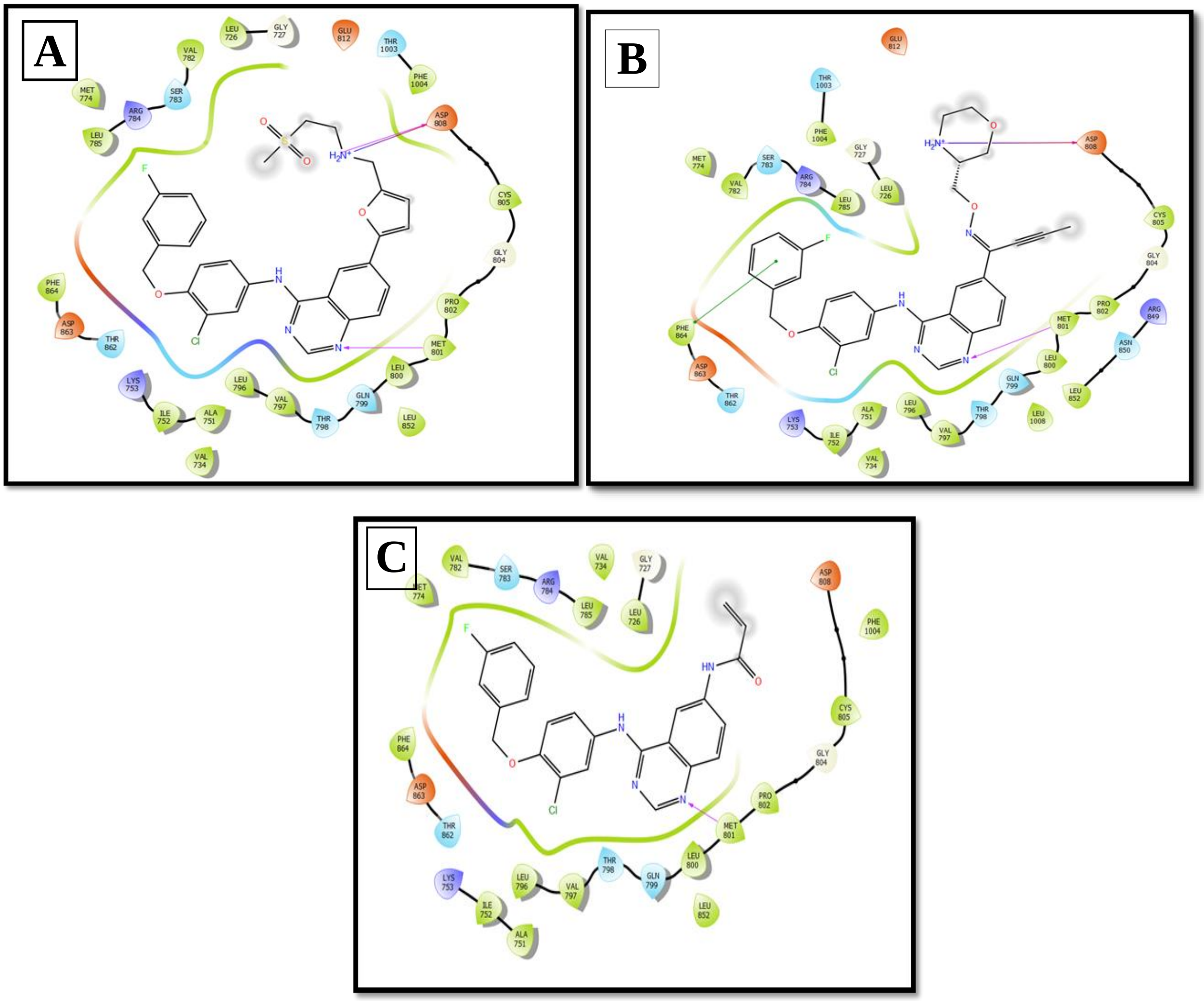

Fig. S1. Binding poses of the top three hits of the training set after docking within HER2 kinase (PDB: 3RCD), showing **(A)** Lapatinib, **(B)** Epertinib and **(C)** Allitinib. Purple arrows indicate hydrogen bonds; red lines represent salt bridges and green lines indicate π-π stacking interactions.

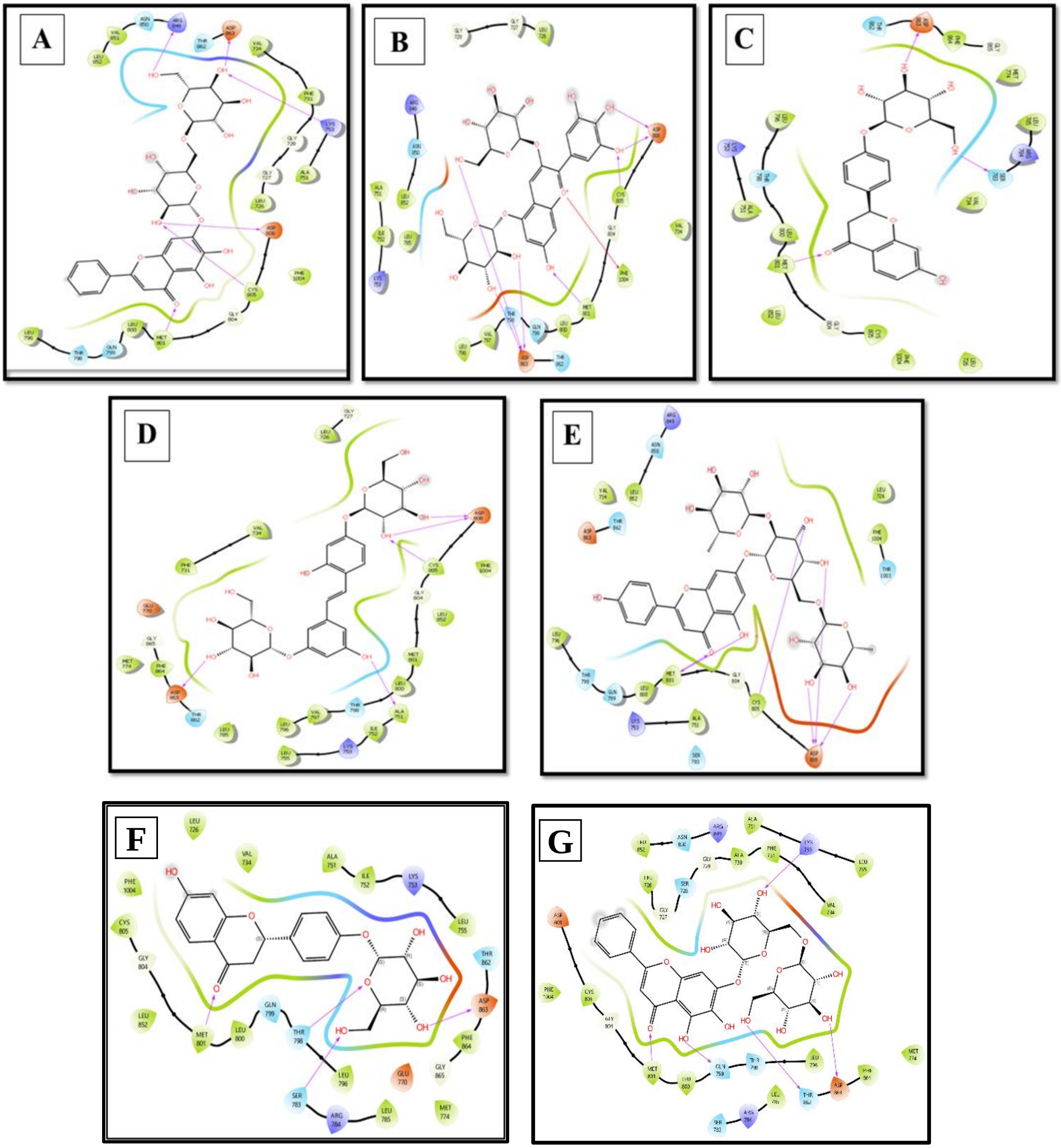

Fig. S2. Two-dimensional Binding poses of **(A)** Oroxin B, **(B)** Delphin, **(C)** Liquiritin, **(D)** Mullberroside A **(E)** Ligustroflavone after Rigid docking in the HER2 kinase (PDB: 3RCD), **(F)** Liquiritin, **(G)** Oroxin B after induced fit docking in the HER2 kinase. Purple arrows represent hydrogen bonds and red lines represent salt bridges.

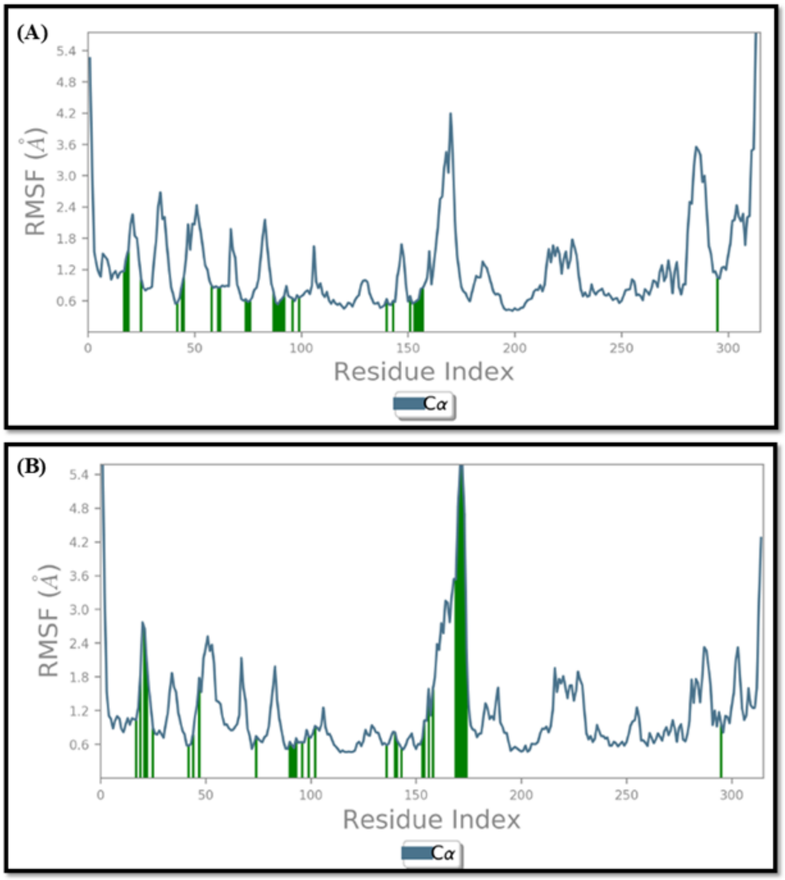

Fig. S3. The RMSF of the HER2 protein for two complexes with **(A)** Liquiritin and **(B)** Oroxin B. Vertical bars with green color indicate protein residues that have interactions with the ligand.

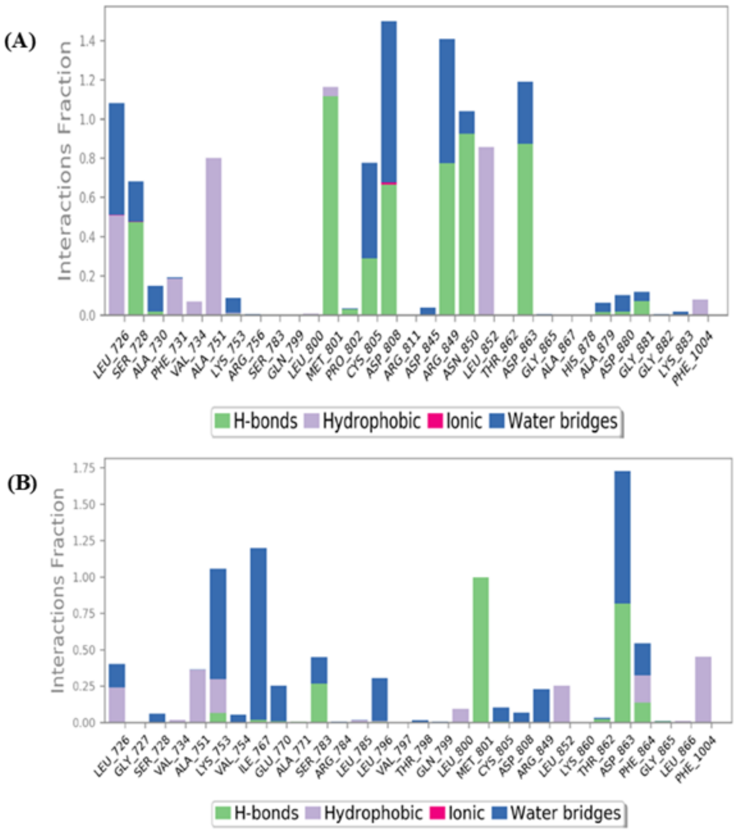

Fig. S4. Histogram depicting the binding interactions between HER2 and its ligand during the simulation time of 300 ns for **(A)** Oroxin B and **(B)** Liquiritin.

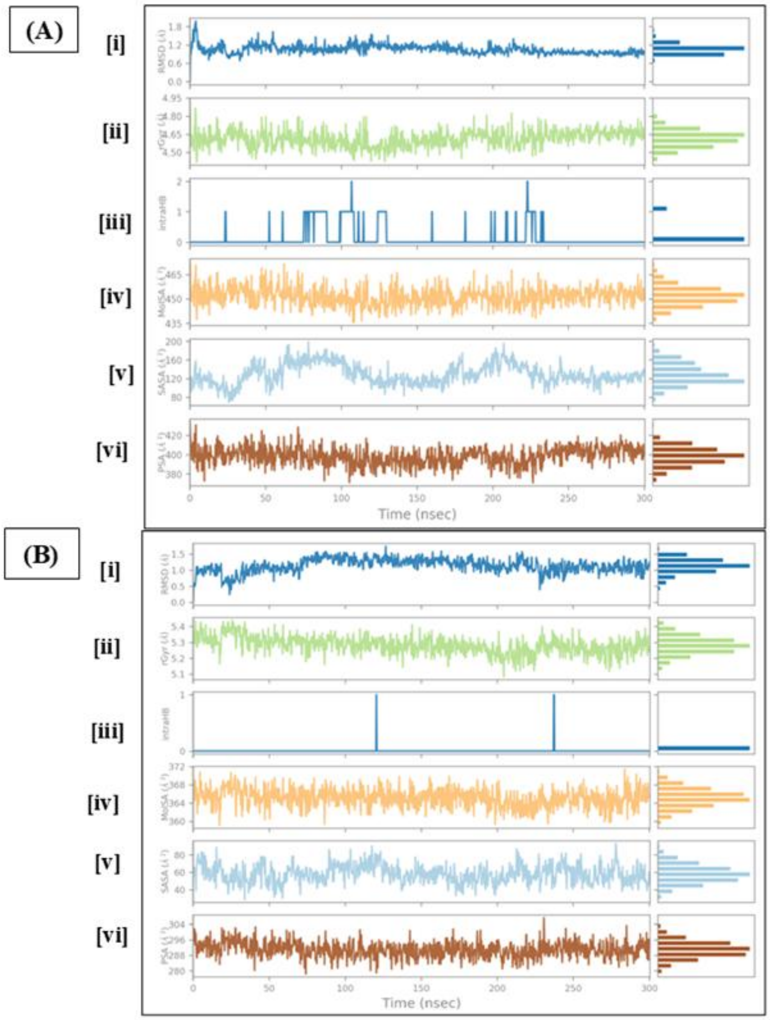

Fig. S5. Study of ligand characteristics during the simulation period of 300 ns for **(A)** Oroxin B and **(B)** Liquiritin. [i]: RMSD, [ii]: rGYr, [iii]: intraHB, [iv]: MolSA, [V]: SASA, [vi]: PSA.

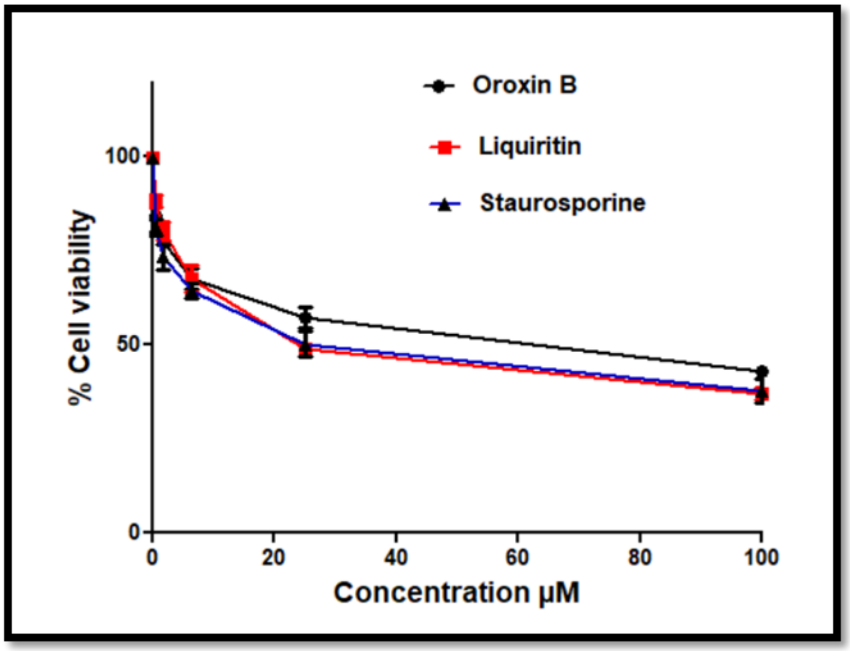

Fig. S6. Dose response curve of Oroxin B and Liquiritin in non-tumorigenic human mammary epithelial MCF10A cell line. Staurosporine is positive control; Error bars represent the SD.

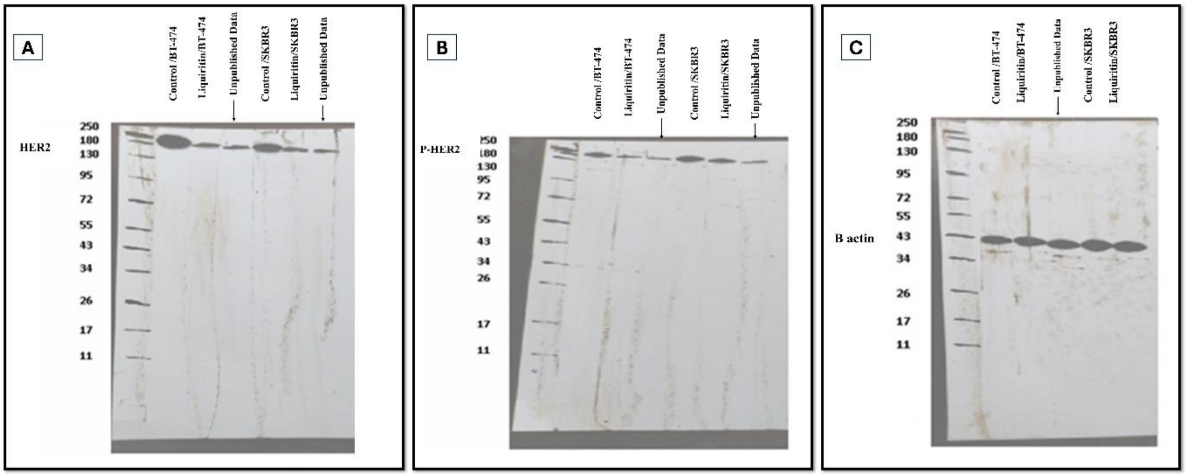

Fig. S7. Raw images of western blot for BT-474 and SKBR3 cell lines representing summary densitometric data for **(A)** HER2, **(B)** P-HER2, **(C)** β-actin.

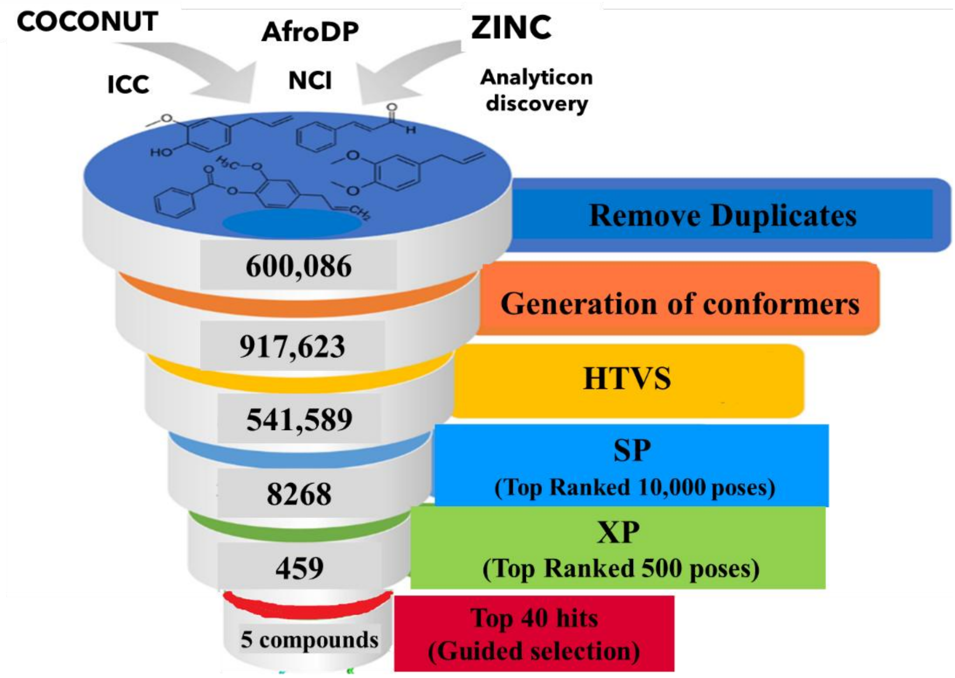

Scheme S1. Summary of the virtual screening campaign of the compiled natural products library against HER2 kinase

References

1 Ishikawa, T. *et al.* Design and synthesis of novel human epidermal growth factor receptor 2 (HER2)/epidermal growth factor receptor (EGFR) dual inhibitors bearing a pyrrolo[3,2-d]pyrimidine scaffold. *J Med Chem* **54**, 8030-8050, doi:10.1021/jm2008634 (2011).

2 Schroeder, R. L., Stevens, C. L. & Sridhar, J. Small molecule tyrosine kinase inhibitors of ErbB2/HER2/Neu in the treatment of aggressive breast cancer. *Molecules* **19**, 15196-15212, doi:10.3390/molecules190915196 (2014).

3 Moulder, S. L. *et al.* Epidermal growth factor receptor (HER1) tyrosine kinase inhibitor ZD1839 (Iressa) inhibits HER2/neu (erbB2)-overexpressing breast cancer cells in vitro and in vivo. *Cancer Res* **61**, 8887-8895 (2001).

4 Ashtekar, S. S., Bhatia, N. M. & Bhatia, M. S. Exploration of Leads from Natural Domain Targeting HER2 in Breast Cancer: An In-Silico Approach. *International Journal of Peptide Research and Therapeutics* **25**, 659-667, doi:10.1007/s10989-018-9712-y (2019).

5 Macpherson, I. R. *et al.* A phase I/II study of epertinib plus trastuzumab with or without chemotherapy in patients with HER2-positive metastatic breast cancer. *Breast Cancer Research* **22**, 1, doi:10.1186/s13058-019-1178-0 (2019).

6 Schlam, I. & Swain, S. M. HER2-positive breast cancer and tyrosine kinase inhibitors: the time is now. *NPJ breast cancer* **7**, 56, doi:10.1038/s41523-021-00265-1 (2021).

7 Meric-Bernstam, F. *et al.* Advances in HER2-Targeted Therapy: Novel Agents and Opportunities Beyond Breast and Gastric Cancer. *Clinical cancer research : an official journal of the American Association for Cancer Research* **25**, 2033-2041, doi:10.1158/1078-0432.ccr-18-2275 (2019).

8 Roy, V. & Perez, E. A. Beyond trastuzumab: small molecule tyrosine kinase inhibitors in HER-2-positive breast cancer. *The oncologist* **14**, 1061-1069, doi:10.1634/theoncologist.2009-0142 (2009).

9 Li, X. *et al.* Discovery and development of pyrotinib: A novel irreversible EGFR/HER2 dual tyrosine kinase inhibitor with favorable safety profiles for the treatment of breast cancer. *European journal of pharmaceutical sciences : official journal of the European Federation for Pharmaceutical Sciences* **110**, 51-61, doi:10.1016/j.ejps.2017.01.021 (2017).

10 Liang, Y., Zhang, T. & Zhang, J. Natural tyrosine kinase inhibitors acting on the epidermal growth factor receptor: Their relevance for cancer therapy. *Pharmacol Res* **161**, 105164, doi:10.1016/j.phrs.2020.105164 (2020).
